# Global estimates on the number of people blind or visually impaired by cataract: a meta-analysis from 2000 to 2020

**DOI:** 10.1038/s41433-024-02961-1

**Published:** 2024-03-09

**Authors:** Vision Loss Expert Group of the Global Burden of Disease Study, Konrad Pesudovs, Van Charles Lansingh, John H. Kempen, Ian Tapply, Arthur G. Fernandes, Maria Vittoria Cicinelli, Alessandro Arrigo, Nicolas Leveziel, Serge Resnikoff, Hugh R. Taylor, Tabassom Sedighi, Seth Flaxman, Mukkharram M. Bikbov, Tasanee Braithwaite, Alain Bron, Ching-Yu Cheng, Monte A. Del Monte, Joshua R. Ehrlich, Leon B. Ellwein, David Friedman, João M. Furtado, Gus Gazzard, Ronnie George, M. Elizabeth Hartnett, Jost B. Jonas, Rim Kahloun, Moncef Khairallah, Rohit C. Khanna, Janet Leasher, Julie-Anne Little, Vinay Nangia, Michal Nowak, Tunde Peto, Pradeep Ramulu, Fotis Topouzis, Mitiadis Tsilimbaris, Ya Xing Wang, Ningli Wang, Rupert Bourne, GBD 2019 Blindness and Vision Impairment Collaborators, Konrad Pesudovs, Van Charles Lansingh, John H. Kempen, Ian Tapply, Arthur G. Fernandes, Maria Vittoria Cicinelli, Alessandro Arrigo, Nicolas Leveziel, Paul Svitil Briant, Theo Vos, Serge Resnikoff, Seth Flaxman, Yohannes Habtegiorgis Abate, Mohammad Abdollahi, Mozhan Abdollahi, Ayele Mamo Abebe, Olumide Abiodun, Richard Gyan Aboagye, Woldu Aberhe Abrha, Hasan Abualruz, Hiwa Abubaker Ali, Eman Abu-Gharbieh, Salahdein Aburuz, Tadele Girum Girum Adal, Mesafint Molla Adane, Isaac Yeboah Addo, Qorinah Estiningtyas Sakilah Adnani, Muhammad Sohail Afzal, Shahin Aghamiri, Bright Opoku Ahinkorah, Aqeel Ahmad, Sajjad Ahmad, Ali Ahmadi, Ayman Ahmed, Haroon Ahmed, Ahmad Samir Alfaar, Abid Ali, Syed Shujait Shujait Ali, Awais Altaf, Hubert Amu, Sofia Androudi, Rodrigo Anguita, Abhishek Anil, Saeid Anvari, Anayochukwu Edward Anyasodor, Francis Appiah, Jalal Arabloo, Mosab Arafat, Damelash Areda, Reza Arefnezhad, Brhane Berhe Aregawi, Akeza Awealom Asgedom, Tahira Ashraf, Seyyed Shamsadin Athari, Bantalem Tilaye Tilaye Atinafu, Maha Moh’d Wahbi Atout, Alok Atreya, Haleh Ayatollahi, Ahmed Y. Azzam, Hassan Babamohamadi, Sara Bagherieh, Yogesh Bahurupi, Atif Amin Baig, Biswajit Banik, Mainak Bardhan, Saurav Basu, Kavita Batra, Nebiyou Simegnew Bayileyegn, Fatemeh Bazvand, Addisu Shunu Beyene, Devidas S. Bhagat, Akshaya Srikanth Bhagavathula, Pankaj Bhardwaj, Sonu Bhaskar, Jasvinder Singh Bhatti, Mukharram Bikbov, Niloufar Bineshfar, Marina G. Birck, Veera R. Bitra, Tasanee Braithwaite, Katrin Burkart, Yasser Bustanji, Zahid A. Butt, Florentino Luciano Caetano dos Santos, Luis Alberto Cámera, Vera L. A. Carneiro, Muthia Cenderadewi, Eeshwar K. Chandrasekar, Vijay Kumar Chattu, Nitin Chitranshi, Hitesh Chopra, Dinh-Toi Chu, Kaleb Coberly, João M. Coelho, Natália Cruz-Martins, Omid Dadras, Xiaochen Dai, Subasish Das, Ana Maria Dascalu, Mohsen Dashti, Maedeh Dastmardi, Berecha Hundessa Demessa, Biniyam Demisse, Diriba Dereje, Awoke Masrie Asrat Derese, Nikolaos Dervenis, Vinoth Gnana Chellaiyan Devanbu, Thanh Chi Do, Thao Huynh Phuong Do, Francisco Winter dos Santos Figueiredo, Arkadiusz Marian Dziedzic, Hisham Atan Edinur, Ferry Efendi, Joshua R. Ehrlich, Michael Ekholuenetale, Temitope Cyrus Ekundayo, Iman El Sayed, Muhammed Elhadi, Mohammad Hassan Emamian, Mehdi Emamverdi, Azin Etemadimanesh, Adeniyi Francis Fagbamigbe, Ayesha Fahim, Hossein Farrokhpour, Ali Fatehizadeh, Alireza Feizkhah, Lorenzo Ferro Desideri, Getahun Fetensa, Florian Fischer, Ali Forouhari, Matteo Foschi, Kayode Raphael Fowobaje, Abhay Motiramji Gaidhane, Aravind P. Gandhi, Miglas W. W. Gebregergis, Mesfin Gebrehiwot, Brhane Gebremariam, Urge Gerema, Fariba Ghassemi, Sherief Ghozy, Mahaveer Golechha, Pouya Goleij, Bárbara Niegia Garcia Goulart, Shi-Yang Guan, Zewdie Gudisa, Sapna Gupta, Veer Bala Gupta, Vivek Kumar Gupta, Arvin Haj-Mirzaian, Aram Halimi, Shahin Hallaj, Samer Hamidi, Mehdi Harorani, Hamidreza Hasani, Demisu Zenbaba Heyi, Nguyen Quoc Hoan, Ramesh Holla, Sung Hwi Hong, Mehdi Hosseinzadeh, Chengxi Hu, John J. Huang, Hong-Han Huynh, Segun Emmanuel Ibitoye, Irena M. Ilic, Mustapha Immurana, Md. Rabiul Islam, Sheikh Mohammed Shariful Islam, Chidozie C. D. Iwu, Louis Jacob, Ammar Abdulrahman Jairoun, Manthan Dilipkumar Janodia, Shubha Jayaram, Har Ashish Jindal, Mohammad Jokar, Nitin Joseph, Charity Ehimwenma Joshua, Vidya Kadashetti, Laleh R. Kalankesh, Rohollah Kalhor, Sagarika Kamath, Himal Kandel, Rami S. Kantar, Ibraheem M. Karaye, Hengameh Kasraei, Soujanya Kaup, Navjot Kaur, Rimple Jeet Kaur, Gbenga A. Kayode, Yousef Saleh Khader, Himanshu Khajuria, Rovshan Khalilov, Mahalaqua Nazli Khatib, Adnan Kisa, Soewarta Kosen, Ai Koyanagi, Kewal Krishan, Mukhtar Kulimbet, Nithin Kumar, Om P. Kurmi, Chandrakant Lahariya, Tuo Lan, Iván Landires, Janet L. Leasher, Munjae Lee, Seung Won Lee, Wei-Chen Lee, Stephen S. Lim, Julie-Anne Little, Preetam Bhalchandra Mahajan, Sandeep B. Maharaj, Alireza Mahmoudi, Razzagh Mahmoudi, Kashish Malhotra, Tauqeer Hussain Mallhi, Vahid Mansouri, Emmanuel Manu, Roy Rillera Marzo, Andrea Maugeri, Colm McAlinden, Wondwosen Mebratu, Tesfahun Mekene Meto, Yang Meng, Abera M. Mersha, Tomislav Mestrovic, Le Huu Nhat Minh, Awoke Misganaw, Manish Mishra, Sanjeev Misra, Nouh Saad Mohamed, Soheil Mohammadi, Mustapha Mohammed, Hoda Mojiri-forushani, Ali H. Mokdad, Hossein Molavi Vardanjani, Mohammad Ali Moni, Fateme Montazeri, Maryam Moradi, Rohith Motappa, Parsa Mousavi, Admir Mulita, Christopher J. L. Murray, Ganesh R. Naik, Gurudatta Naik, Shumaila Nargus, Zuhair S. Natto, Biswa Prakash Nayak, Mohammad Negaresh, Hadush Negash, Dang H. Nguyen, Phat Tuan Nguyen, Van Thanh Nguyen, Robina Khan Niazi, Osaretin Christabel Okonji, Andrew T. Olagunju, Matthew Idowu Olatubi, Michal Ordak, Uchechukwu Levi Osuagwu, Nikita Otstavnov, Mayowa O. Owolabi, Jagadish Rao Padubidri, Ashok Pandey, Georgios D. Panos, Shahina Pardhan, Seoyeon Park, Jay Patel, Shrikant Pawar, Prince Peprah, Ionela-Roxana Petcu, Alireza Peyman, Hoang Tran Pham, Mohsen Pourazizi, Nguyen Khoi Quan, Fakher Rahim, Vafa Rahimi-Movaghar, Mohammad Hifz Ur Rahman, Sathish Rajaa, Shakthi Kumaran Ramasamy, Premkumar Ramasubramani, Shubham Ranjan, Mohammad-Mahdi Rashidi, Rama Shankar Rath, Annisa Utami Rauf, Salman Rawaf, Amirmasoud Rayati Damavandi, Elrashdy Moustafa Mohamed Redwan, Priyanka Roy, Koushik Roy Pramanik, Zahra Saadatian, Siamak Sabour, Basema Saddik, Umar Saeed, Sare Safi, Sher Zaman Safi, Amene Saghazadeh, Fatemeh Saheb Sharif-Askari, Amirhossein Sahebkar, Mohammad Ali Sahraian, Joseph W. Sakshaug, Mohamed A. Saleh, Sara Samadzadeh, Yoseph Leonardo Samodra, Vijaya Paul Samuel, Abdallah M. Samy, Aswini Saravanan, Siddharthan Selvaraj, Farbod Semnani, Sabyasachi Senapati, Yashendra Sethi, Seyed Arsalan Seyedi, Allen Seylani, Amira A. Shaheen, Samiah Shahid, Moyad Jamal Shahwan, Masood Ali Shaikh, Sunder Sham, Muhammad Aaqib Shamim, Mohammed Shannawaz, Bereket Beyene Shashamo, Maryam Shayan, Aminu Shittu, Ivy Shiue, K. M. Shivakumar, Seyed Afshin Shorofi, Migbar Mekonnen Sibhat, Emmanuel Edwar Siddig, Juan Carlos Silva, Jasvinder A. Singh, Paramdeep Singh, Eirini Skiadaresi, Yonatan Solomon, Raúl A. R. C. Sousa, Chandrashekhar T. Sreeramareddy, Vladimir I. Starodubov, Mohana Devi Subramaniam, Sri Susanty, Seyyed Mohammad Tabatabaei, Birhan Tsegaw Taye, Gebrehiwot Teklay, Mohamad-Hani Temsah, Dufera Rikitu Terefa, Jansje Henny Vera Ticoalu, Temesgen Mohammed Toma, Aristidis Tsatsakis, Guesh Mebrahtom Tsegay, Munkhtuya Tumurkhuu, Biruk Shalmeno Tusa, Sree Sudha Ty, Chukwudi S. Ubah, Muhammad Umair, Tungki Pratama Umar, Rohollah Valizadeh, Jef Van den Eynde, Stephanie Louise Watson Watson, Tewodros Eshete Wonde, Guadie Sharew Wondimagegn, Hong Xiao, Yao Yao, Iman Yazdani Nia, Arzu Yiğit, Yazachew Yismaw, Dong Keon Yon, Naohiro Yonemoto, Yuyi You, Chuanhua Yu, Mikhail Sergeevich Zastrozhin, Hanqing Zhao, Makan Ziafati, Magdalena Zielińska, Yossef Teshome Zikarg, Mohammad Zoladl, Jaimie D. Steinmetz

**Affiliations:** 1 Medicine & Health, University of New South Wales, Sydney, NSW Australia; 2 HelpMeSee, Instituto Mexicano de Oftalmologia, Santiago de Querétaro, Mexico; 3 University of Miami, Gables, USA; 4 University of Utah, Salt Lake City, USA; 5 Department of Ophthalmology, Massachusetts Eye and Ear/Shepens Eye Research Institute/Harvard Medical School, Boston, MA USA; 6 Eye Unit, MyungSung Christian Medical Center (MCM) Comprehensive Specialized Hospital and MyungSun Medical College, Addis Ababa, Ethiopia; 7 Department of Ophthalmology, Addis Ababa University, Addis Ababa, Ethiopia; 8 Sight for Souls, Bellevue, WA USA; 9 Addenbrooke’s Hospital, Cambridge, UK; 10 Federal University of Sao Paolo, Sao Paolo, SP Brazil; 11 University of Calgary, Calgary, AB Canada; 12 School of Medicine, Vita-Salute San Raffaele University, Milan, Italy; 13 Department of Ophthalmology, IRCCS San Raffaele Scientific Institute, Milan, Italy; 14 IRCCS San Raffaele Scientific Institute, Vita-Salute University, Milan, Italy; 15 University of Poitiers, Poitiers, France; 16 CHU de Poitiers, Poitiers, France; 17 Brien Holden Vision Institute, Sydney, NSW Australia; 18 School of Optometry and Vision Sciences, Faculty of Medicine, University of New South Wales, Sydney, NSW Australia; 19 School of Population and Global Health, University of Melbourne, Carlton, VIC Australia; 20 Vision and Eye Research Institute, Anglia Ruskin University, Cambridge, UK; 21 Department of Computer Science, University of Oxford, Oxford, UK; 22 Ufa Eye Research Institute, Ufa, Russia; 23 School of Life Course and Population Sciences, King’s College London, London, UK; 24 The Medical Eye Unit, Guy’s and St Thomas’ NHS Foundation Trust, London, UK; 25 University Hospital, Dijon, France; 26 National University of Singapore, Singapore, Singapore; 27 Singapore Eye Research Institute, Singapore, Singapore; 28 University of Michigan, Ann Arbor, USA; 29 Kellogg Eye Center, Ann Arbor, USA; 30 Institute for Social Research, University of Michigan, Ann Arbor, USA; 31 Department of Ophthalmology and Visual Sciences, University of Michigan, Ann Arbor, USA; 32 National Eye Institute, Bethesda, MD USA; 33 Mass Eye and Ear, Harvard Medical School, Boston, USA; 34 Ribeirão Preto Medical School, University of São Paulo, Sao Paulo, Brazil; 35 Institute of Ophthalmology UCL & NIHR Biomedical Research Centre, London, UK; 36 Sankara Nethralaya, Medical Research Foundation, Chennai, 600006 India; 37 Stanford University, Stanford, USA; 38 Department of Ophthalmology, Medical Faculty Mannheim, Heidelberg University, Heidelberg, Germany; 39 Associated Ophthalmologists of Monastir, Monastir, Tunisia; 40 Fattouma Bourguiba University Hospital, University of Monastir, Monastir, Tunisia; 41 Allen Foster Community Eye Health Research Centre, Gullapalli Pratibha Rao International Centre for Advancement of Rural Eye care, L.V. Prasad Eye Institute, Hyderabad, India; 42 Brien Holden Eye Research Centre, L.V. Prasad Eye Institute, Banjara Hills, Hyderabad, India; 43 School of Optometry and Vision Science, University of New South Wales, Sydney, Australia; 44 University of Rochester, School of Medicine and Dentistry, Rochester, NY USA; 45 Nova Southeastern University College for Optometry, Fort Lauderdale, Florida USA; 46 Ulster University, Coleraine, UK; 47 Suraj Eye Institute, Nagpur, India; 48 Institute of Optics and Optometry, University of Social Science, 121 Gdanska str, Lodz, 90-519 Poland; 49 Centre for Public Health, Queens University Belfast, Northern Ireland, Belfast, UK; 50 John Hopkins Wilmer Eye Institute, Baltimore, USA; 51 1st Department of Ophthamology, Medical School, Aristotle University of Thessaloniki, Ahepa Hospital, Thessaloniki, Greece; 52 University of Crete Medical School, Giofirakia, Greece; 53 Beijing Institute of Ophthamology, Beijing Tongren Hospital, Capital Medical University, Beijing Ophthamology and Visual Sciences Key Laboratory, Beijing, China; 54 Beijing Institute of Ophthamology, Beijing Tongren Eye Center, Beijing Tongren Hospital, Capital Medical University, Beijing, China; 55 School of Optometry and Vision Science, University of New South Wales, Sydney, NSW Australia; 56 Chief Medical Office, HelpMeSee, New York, NY USA; 57 Mexican Institute of Ophthalmology, Queretaro, Mexico; 58 Department of Ophthalmology, Harvard University, Boston, MA USA; 59 Eye Unit, MyungSung Medical College, Addis Ababa, Ethiopia; 60 Department of Ophthalmology, Cambridge University Hospitals, Cambridge, UK; 61 Department of Ophthalmology and Visual Sciences, Federal University of São Paulo, Sao Paulo, Brazil; 62 Department of Ophthalmology, San Raffaele Scientific Institute, Milano, Italy; 63 Scientific Institute San Raffaele Hospital, Vita-Salute University, Milan, Italy; 64 Ophthalmology Department, CHU de Poitiers (Poitiers University Hospital), Poitiers, France; 65 Unité 1084, National Institute of Health and Medical Research (INSERM), Poitiers, France; 66 Institute for Health Metrics and Evaluation, University of Washington, Seattle, WA USA; 67 Department of Health Metrics Sciences, School of Medicine, University of Washington, Seattle, WA USA; 68 Department of Mathematics, Imperial College London, London, UK; 69 Department of Clinical Governance and Quality Improvement, Aleta Wondo Hospital, Aleta Wondo, Ethiopia; 70 The Institute of Pharmaceutical Sciences (TIPS), Tehran University of Medical Sciences, Tehran, Iran; 71 School of Pharmacy, Tehran University of Medical Sciences, Tehran, Iran; 72 School of Medicine, Shiraz University of Medical Sciences, Shiraz, Iran; 73 Pediatrics Nursing Department, Debre Berhan University, Debre Berhan, Ethiopia; 74 Department of Community Medicine, Babcock University, Ilishan-Remo, Nigeria; 75 Department of Family and Community Health, University of Health and Allied Sciences, Ho, Ghana; 76 Department of Adult Health Nursing, Aksum University, Aksum, Ethiopia; 77 Department of Nursing, Al Zaytoonah University of Jordan, Amman, Jordan; 78 Department of Banking and Finance, University of Human Development, Sulaymaniyah, Iraq; 79 Clinical Sciences Department, University of Sharjah, Sharjah, United Arab Emirates; 80 Department of Therapeutics, United Arab Emirates University, Al Ain, United Arab Emirates; 81 College of Pharmacy, University of Jordan, Amman, Jordan; 82 Department of Public Health, Wolkite University, Wolkite, Ethiopia; 83 College of Medicine and Health Sciences, Bahir Dar University, Bahir Dar, Ethiopia; 84 Centre for Social Research in Health, University of New South Wales, Sydney, NSW Australia; 85 Quality and Systems Performance Unit, Cancer Institute NSW, Sydney, NSW Australia; 86 Faculty of Medicine, Universitas Padjadjaran (Padjadjaran University), Bandung, Indonesia; 87 Department of Life Sciences, University of Management and Technology, Lahore, Pakistan; 88 Department of Biotechnology, Shahid Beheshti University of Medical Sciences, Tehran, Iran; 89 School of Public Health, University of Technology Sydney, Sydney, NSW Australia; 90 Department of Medical Biochemistry, Shaqra University, Shaqra, Saudi Arabia; 91 Department of Health and Biological Sciences, Abasyn University, Peshawar, Pakistan; 92 Department of Natural Sciences, Labanese American University, Beirut, Lebanon; 93 Department of Epidemiology and Biostatistics, Shahrekord University of Medical Sciences, Shahrekord, Iran; 94 Department of Epidemiology, Shahid Beheshti University of Medical Sciences, Tehran, Iran; 95 Institute of Endemic Diseases, University of Khartoum, Khartoum, Sudan; 96 Swiss Tropical and Public Health Institute, University of Basel, Basel, Switzerland; 97 Department of Biosciences, COMSATS Institute of Information Technology, Islamabad, Pakistan; 98 Department of Ophthalmology, University of Leipzig Medical Center, Leipzig, Germany; 99 Department of Ophthalmology, Charité Universitätsmedizin Berlin (Charité Medical University Berlin), Berlin, Germany; 100 Department of Zoology, Abdul Wali Khan University Mardan, Mardan, Pakistan; 101 Center for Biotechnology and Microbiology, University of SWAT, Swat, Pakistan; 102 Centre for Research in Molecular Medicine, Institute of Molecular Biology and Biotechnology, Lahore, Pakistan; 103 Department of Population and Behavioural Sciences, University of Health and Allied Sciences, Ho, Ghana; 104 Department of Medicine, University of Thessaly, Volos, Greece; 105 Department of Ophthalmology, Inselspital, Bern, Switzerland; 106 Department of Vitreoretinal, Moorfields Eye Hospital, London, UK; 107 Department of Pharmacology, All India Institute of Medical Sciences, Jodhpur, India; 108 All India Institute of Medical Sciences, Bhubaneswar, India; 109 Regenerative Medicine, Organ Procurement and Transplantation Multi-diciplinary Center, Guilan University of Medical Sciences, Rasht, Iran; 110 School of Dentistry and Medical Sciences, Charles Sturt University, Orange, NSW Australia; 111 Department of Social Sciences, Berekum College of Education, Berekum, Ghana; 112 School of Public Health, Kwame Nkrumah University of Science and Technology, Kumasi, Ghana; 113 Health Management and Economics Research Center, Iran University of Medical Sciences, Tehran, Iran; 114 College of Pharmacy, Al Ain University, Abu Dhabi, United Arab Emirates; 115 College of Art and Science, Ottawa University, Surprise, AZ USA; 116 School of Life Sciences, Arizona State University, Tempe, AZ USA; 117 Department of Anatomy, Shiraz University of Medical Sciences, Shiraz, Iran; 118 College of Medicine and Health Sciences, Adigrat University, Adigrat, Ethiopia; 119 Department of Environmental Health, Mekelle University, Mekelle, Ethiopia; 120 University Institute of Radiological Sciences and Medical Imaging Technology, The University of Lahore, Lahore, Pakistan; 121 Department of Immunology, Zanjan University of Medical Sciences, Zanjan, Iran; 122 School of Nursing and Midwifery, Debre Berhan University, Debre Berhan, Ethiopia; 123 Faculty of Nursing, Philadelphia University, Amman, Jordan; 124 Department of Forensic Medicine, Lumbini Medical College, Palpa, Nepal; 125 Department of Health Information Management, Iran University of Medical Sciences, Tehran, Iran; 126 Department of Neurovascular Research, Nested Knowledge, Inc, Saint Paul, MN USA; 127 Faculty of Medicine, October 6 University, 6th of October City, Egypt; 128 Department of Nursing, Semnan University of Medical Sciences and Health Services, Semnan, Iran; 129 School of Medicine, Isfahan University of Medical Sciences, Isfahan, Iran; 130 Department of Community Medicine and Family Medicine, All India Institute of Medical Sciences, Rishikesh, India; 131 University Institute of Public Health, The University of Lahore, Lahore, Pakistan; 132 Institute of Health and Wellbeing (IHW), Federation University Australia, Melbourne, VC Australia; 133 Manna Institute, University of New England, Armidale, NSW Australia; 134 Miami Cancer Institute, Baptist Health South Florida, Miami, FL USA; 135 Department of Academics, Indian Institute of Public Health, Gurgaon, India; 136 Department of Medical Education, University of Nevada, Las Vegas, Las Vegas, NV USA; 137 Department of Surgery, Jimma University, jimma, Ethiopia; 138 Farabi Eye Hospital, Tehran University of Medical Sciences, Tehran, Iran; 139 School of Medicine and Public Health, University of Newcastle, Newcastle, NSW Australia; 140 School of Public Health, Haramaya University, Harar, Ethiopia; 141 Department of Forensic Chemistry, Government Institute of Forensic Science, Aurangabad, Aurangabad, India; 142 Department of Public Health, North Dakota State University, Fargo, ND USA; 143 Department of Community Medicine and Family Medicine, All India Institute of Medical Sciences, Jodhpur, India; 144 School of Public Health, All India Institute of Medical Sciences, Jodhpur, India; 145 Global Health Neurology Lab, NSW Brain Clot Bank, Sydney, NSW Australia; 146 Department of Neurology and Neurophysiology, South West Sydney Local Heath District and Liverpool Hospital, Sydney, NSW Australia; 147 Human Genetics and Molecular Medicine, Central University of Punjab, Bathinda, India; 148 Epidemiology Department, Ufa Eye Research Institute, Ufa, Russia; 149 Department of Ophthalmology, Shahid Beheshti University of Medical Sciences, Tehran, Iran; 150 Department of Medicine, Division of Clinical Epidemiology, McGill University, Montreal, QC Canada; 151 Centre for Outcomes Research and Evaluation, Research Institute of the McGill University Health Centre, Montreal, QC Canada; 152 Faculty of Health Sciences, University of Botswana, Gaborone, Botswana; 153 Ophthalmology Department, Moorfields Eye Hospital NHS Foundation Trust, London, UK; 154 International Centre for Eye Health, London School of Hygiene & Tropical Medicine, London, UK; 155 Department of Biopharmaceutics and Clinical Pharmacy, The University of Jordan, Amman, Jordan; 156 Department of Basic Biomedical Sciences, University of Sharjah, Sharjah, United Arab Emirates; 157 School of Public Health and Health Systems, University of Waterloo, Waterloo, ON Canada; 158 Al Shifa School of Public Health, Al Shifa Trust Eye Hospital, Rawalpindi, Pakistan; 159 Harvard Business School, Harvard University, Boston, MA USA; 160 Internal Medicine Department, Hospital Italiano de Buenos Aires (Italian Hospital of Buenos Aires), Buenos Aires, Argentina; 161 Board of Directors, Argentine Society of Medicine, Buenos Aires, Argentina; 162 School of Sciences, University of Minho, Braga, Portugal; 163 Association of Licensed Optometry Professionals, Linda-a-Velha, Portugal; 164 College of Public Health, Medical and Veterinary Sciences, James Cook University, Townsville, Australia; 165 Public Health Departement, University of Mataram, Mataram, Indonesia; 166 Department of Anesthesiology and Perioperative Medicine, University of Rochester, Rochester, NY USA; 167 Temerty Faculty of Medicine, University of Toronto, Toronto, ON Canada; 168 Saveetha Dental College, Saveetha University, Chennai, India; 169 Macquarie Medical School, Macquarie University, Sydney, NSW Australia; 170 Chitkara College of Pharmacy, Chitkara Univeristy, Punjab, India; 171 Center for Biomedicine and Community Health, VNU-International School, Hanoi, Viet Nam; 172 University Hospital Center of Porto, University of Porto, Porto, Portugal; 173 Therapeutic and Diagnostic Technologies, Cooperativa de Ensino Superior Politécnico e Universitário (Polytechnic and University Higher Education Cooperative), Gandra, Portugal; 174 Institute for Research and Innovation in Health, University of Porto, Porto, Portugal; 175 Department of Addiction Medicine, Haukland University Hospital, Bergen, Norway; 176 Department of Global Public Health and Primary Care, University of Bergen, Bergen, Norway; 177 Ingram School of Engineering, Texas State University, San Marcos, TX USA; 178 Ophthalmology Department, Carol Davila University of Medicine and Pharmacy, Bucharest, Romania; 179 Ophthalmology Department, Emergency University Hospital Bucharest, Bucuresti, Romania; 180 Department of Radiology, Tabriz University of Medical Sciences, Tabriz, Iran; 181 Iran University of Medical Sciences, Tehran, Iran; 182 Tehran University of Medical Science, Tehran University of Medical Sciences, Tehran, Iran; 183 USAID-JSI, Jimma University, Addis Ababa, Ethiopia; 184 Department of Nursing, Arba Minch University, Arba Minch, Ethiopia; 185 Department of Biomedical Sciences, Jimma University, Jimma, Ethiopia; 186 St Paul’s Eye Unit, Royal Liverpool University Hospital, Liverpool, UK; 187 Department of Ophthalmology, Aristotle University of Thessaloniki, Thessaloniki, Greece; 188 Department of Community Medicine, Chettinad Hospital and Research Institute, Chennai, India; 189 Department of Medicine, Pham Ngoc Thach University of Medicine, Ho Chi Minh City, Viet Nam; 190 Department of Medicine, Can Tho University of Medicine and Pharmacy, Can Tho, Viet Nam; 191 Epidemiology and Data Analysis Laboratory, University Center FMABC, Santo André, Brazil; 192 Department of Conservative Dentistry with Endodontics, Medical University of Silesia, Katowice, Poland; 193 School of Health Sciences, Universiti Sains Malaysia (University of Science Malaysia), Kubang Kerian, Malaysia; 194 Advanced Nursing Department, Universitas Airlangga, Surabaya, Indonesia; 195 Department of Ophthalmology and Visual Sciences, University of Michigan, Ann Arbor, GA USA; 196 Institute for Health Care Policy and Innovation, University of Michigan, Ann Arbor, MI USA; 197 Department of Epidemiology and Medical Statistics, University of Ibadan, Ibadan, Nigeria; 198 Faculty of Public Health, University of Ibadan, Ibadan, Nigeria; 199 Department of Biological Sciences, University of Medical Sciences, Ondo, Ondo, Nigeria; 200 Biomedical Informatics and Medical Statistics Department, Alexandria University, Alexandria, Egypt; 201 Faculty of Medicine, University of Tripoli, Tripoli, Libya; 202 Ophthalmic Epidemiology Research Center, Shahroud University of Medical Sciences, Shahroud, Iran; 203 Department of Ophthalmology, University of California Los Angeles, Los Angeles, CA USA; 204 Department of Physical Medicine and Rehabilitation, Johns Hopkins University, Baltimore, MD USA; 205 Research Centre for Healthcare and Community, Coventry University, Coventry, UK; 206 Department of Oral Biology, The University of Lahore, Lahore, Pakistan; 207 School of Medicine, Tehran University of Medical Sciences, Tehran, Iran; 208 Endocrinology and Metabolism Research Institute, Non-Communicable Diseases Research Center (NCDRC), Tehran, Iran; 209 Department of Environmental Health Engineering, Isfahan University of Medical Sciences, Isfahan, Iran; 210 Department of Social Medicine and Epidemiology, Guilan University of Medical Sciences, Rasht, Iran; 211 University Eye Clinic, University of Genoa, Genoa, Italy; 212 Department of Nursing, Wollega University, Nekemte, Ethiopia; 213 Institute of Public Health, Charité Universitätsmedizin Berlin (Charité Medical University Berlin), Berlin, Germany; 214 Department of Ophthalmology, Isfahan University of Medical Sciences, Isfahan, Iran; 215 Emergency Department, Isfahan University of Medical Sciences, Isfahan, Iran; 216 Department of Biotechnological and Applied Clinical Sciences (DISCAB), Multiple Sclerosis Research Center, L’Aquila, Italy; 217 Department of Neuroscience, Multiple Sclerosis Research Center, Ravenna, Italy; 218 Child Survival Unit, Centre for African Newborn Health and Nutrition, Ibadan, Nigeria; 219 Department of Community Medicine, Datta Meghe Institute of Medical Sciences, Wardha, India; 220 Department of Community Medicine, ESIC Medical College & Hospital, Hyderabad, India; 221 Department of Midwifery, Adigrat University, Adigrat, Ethiopia; 222 Department of Environmental Health, Wollo University, Dessie, Ethiopia; 223 Department of Health, Policy Research Institute, Mekelle, Ethiopia; 224 Simon Fraser University, Mekelle, Ethiopia; 225 Department of Public Health, Jimma University, Jimma, Ethiopia; 226 Department of Ophthalmology, Tehran University of Medical Sciences, Tehran, Iran; 227 Department of Radiology, Mayo Clinic, Rochester, MN USA; 228 Health Systems and Policy Research Department, Indian Institute of Public Health, Gandhinagar, India; 229 Department of Genetics, Sana Institute of Higher Education, Sari, Iran; 230 Universal Scientific Education and Research Network (USERN), Kermanshah University of Medical Sciences, Kermanshah, Iran; 231 Postgraduate Program in Epidemiology, Federal University of Rio Grande do Sul, Porto Alegre, Brazil; 232 Department of Epidemiology and Biostatistics, Anhui Medicla University, Hefei, China; 233 Department of Anesthesia, Madda Walabu University, Goba, Ethiopia; 234 Toxicology Department, Shriram Institute for Industrial Research, Delhi, India; 235 School of Medicine, Deakin University, Geelong, VIC Australia; 236 Faculty of Medicine Health and Human Sciences, Macquarie University, Sydney, NSW Australia; 237 Department of Radiology, Massachusetts General Hospital, Boston, MA USA; 238 Obesity Research Center, Shahid Beheshti University of Medical Sciences, Tehran, Iran; 239 Research Institute for Endocrine Sciences, Shahid Beheshti University of Medical Sciences, Tehran, Iran; 240 Department of Ophthalmology, Thomas Jefferson University, Philadelphia, PA USA; 241 Department of Ophthalmology, University of California San Diego, La Jolla, CA USA; 242 School of Health and Environmental Studies, Hamdan Bin Mohammed Smart University, Dubai, United Arab Emirates; 243 Department of Nursing, Arak University of Medical Sciences, Arak, Iran; 244 Department of Ophthalmology, Iran University of Medical Sciences, Karaj, Iran; 245 Department of Public Health, Madda Walabu University, Goba, Ethiopia; 246 School of Dentistry, Hanoi Medical University, Hanoi, Viet Nam; 247 Kasturba Medical College, Mangalore, Manipal Academy of Higher Education, Manipal, India; 248 Department of Pediatrics, Yonsei University College of Medicine, Seoul, South Korea; 249 Research Department, Electronic Medical Records for the Developing World, York, UK; 250 Institute of Research and Development, Duy Tan University, Da Nang, Viet Nam; 251 Department of Computer Science, University of Human Development, Sulaymaniyah, Iraq; 252 Department of Psychology, Tsinghua University, Beijing, China; 253 Department of Ophthalmology and Visual Science, Yale University, New Haven, CT USA; 254 School of Biotechnology, Tan Tao University, Long An, Viet Nam; 255 Department of Health Promotion and Education, University of Ibadan, Ibadan, Nigeria; 256 Faculty of Medicine, University of Belgrade, Belgrade, Serbia; 257 Institute of Health Research, University of Health and Allied Sciences, Ho, Ghana; 258 Department of Pharmacy, University of Asia Pacific, Dhaka, Bangladesh; 259 Institute for Physical Activity and Nutrition, Deakin University, Burwood, VIC Australia; 260 Sydney Medical School, University of Sydney, Sydney, NSW Australia; 261 School of Health Systems and Public Health, University of Pretoria, Pretoria, South Africa; 262 Research and Development Unit, Biomedical Research Networking Center for Mental Health Network (CiberSAM), Sant Boi de Llobregat, Spain; 263 Faculty of Medicine, University of Versailles Saint-Quentin-en-Yvelines, Montigny-le-Bretonneux, France; 264 Department of Clinical Pharmacy, University of Science Malaysia, Penang, Malaysia; 265 Department of Health and Safety, United Arab Emirates University, Dubai, United Arab Emirates; 266 Manipal College of Pharmaceutical Sciences, Manipal Academy of Higher Education, Manipal, India; 267 Department of Biochemistry, Government Medical College, Mysuru, India; 268 National Health System Resource Centre, Ministry of Health & Family Welfare, New Delhi, India; 269 Zoonoses Research Center, Islamic Azad University, Karaj, Iran; 270 Department of Clinical Sciences, Jahrom University of Medical Sciences, Jahrom, Iran; 271 Department of Community Medicine, Manipal Academy of Higher Education, Mangalore, India; 272 Department of Economics, National Open University, Benin City, Nigeria; 273 Department of Oral and Maxillofacial Pathology, Krishna Vishwa Vidyapeeth (Deemed to be University), Karad, India; 274 Social Determinants of Health Research Center, Gonabad University of Medical Sciences, Gonabad, Iran; 275 Institute for Prevention of Non-communicable Diseases, Qazvin University of Medical Sciences, Qazvin, Iran; 276 Health Services Management Department, Qazvin University of Medical Sciences, Qazvin, Iran; 277 Manipal Institute of Management, Manipal Academy of Higher Education, Manipal, India; 278 Save Sight Institute, University of Sydney, Sydney, NSW Australia; 279 Sydney Eye Hospital, South Eastern Sydney Local Health District, Sydney, NSW Australia; 280 The Hansjörg Wyss Department of Plastic and Reconstructive Surgery, Nab’a Al-Hayat Foundation for Medical Sciences and Health Care, New York, NY USA; 281 Department of Cleft Lip and Palate Surgery, Global Smile Foundation, Norwood, MA USA; 282 School of Health Professions and Human Services, Hofstra University, Hempstead, NY USA; 283 Department of Anesthesiology, Montefiore Medical Center, Bronx, NY USA; 284 Eye Research Center, Iran University of Medical Sciences, Tehran, Iran; 285 Health Policy Research Center, Shiraz University of Medical Sciences, Shiraz, Iran; 286 Department of Ophthalmology, Yenepoya Medical College, Mangalore, India; 287 Department of ENT, Dr. B. R. Ambedkar State Institute of Medical Sciences (AIMS), Mohali, India; 288 International Research Center of Excellence, Institute of Human Virology Nigeria, Abuja, Nigeria; 289 Julius Centre for Health Sciences and Primary Care, Utrecht University, Utrecht, Netherlands; 290 Department of Public Health, Jordan University of Science and Technology, Irbid, Jordan; 291 Amity Institute of Forensic Sciences, Amity University, Noida, India; 292 Department of Biophysics and Biochemistry, Baku State University, Baku, Azerbaijan; 293 Azerbaijan State University of Economics (UNEC), Baku, Azerbaijan; 294 Global Consortium for Public Health Research, Datta Meghe Institute of Higher Education and Research, Wardha, India; 295 School of Health Sciences, Kristiania University College, Oslo, Norway; 296 Department of International Health and Sustainable Development, Tulane University, New Orleans, LA USA; 297 Independent Consultant, Jakarta, Indonesia; 298 San Juan de Dios Sanitary Park, Barcelona, Spain; 299 Department of Anthropology, Panjab University, Chandigarh, India; 300 Department of Health Research, Almaty, Kazakhstan; 301 Atchabarov Scientific Research Institute of Fundamental and Aplied Medicine, Kazakh National Medical University, Almaty, Kazakhstan; 302 Faculty of Health and Life Sciences, Coventry University, Coventry, UK; 303 Department of Medicine, McMaster University, Hamilton, ON Canada; 304 Department of Health Policy and Strategy, Foundation for People-centric Health Systems, New Delhi, India; 305 SD Gupta School of Public Health, Indian Institute of Health Management Research University, Jaipur, India; 306 Department of Surgery, Washington University in St. Louis, Saint Louis, MO USA; 307 Unit of Genetics and Public Health, Institute of Medical Sciences, Las Tablas, Panama; 308 Ministry of Health, Herrera, Panama; 309 College of Optometry, Nova Southeastern University, Fort Lauderdale, FL USA; 310 Department of Medical Humanities and Social Medicine, Ajou University School of Medicine, Suwon, South Korea; 311 Medial Research Collaborating Center, Ajou University Medical Center, Suwon, South Korea; 312 Department of Precision Medicine, Sungkyunkwan University, Suwon-si, South Korea; 313 Department of Internal Medicine, University of Texas, Galveston, TX USA; 314 School of Biomedical Sciences, Coleraine, UK; 315 Department of Community Medicine, Jawaharlal Institute of Postgraduate Medical Education and Research, Karaikal, India; 316 School of Pharmacy, University of the West Indies, St. Augustine, Trinidad and Tobago; 317 Fellow, Planetary Health Alliance, Boston, MA USA; 318 Department of Food Hygiene and Safety, Qazvin University of Medical Sciences, Qazvin, Iran; 319 Department of Internal Medicine, Dayanand Medical College and Hospital, Ludhiana, India; 320 Department of Clinical Pharmacy, Jouf University, Sakaka, Saudi Arabia; 321 Digestive Diseases Research Institute, Tehran University of Medical Sciences, Tehran, Iran; 322 Department of Public Health, Management and Science University, Shah Alam, Malaysia; 323 Jeffrey Cheah School of Medicine and Health Sciences, Monash University, Subang Jaya, Malaysia; 324 Department GF Ingrassia, University of Catania, Catania, Italy; 325 Department of Ophthalmology, Princess of Wales Hospital, Wales, UK; 326 School of Optometry and Vision Sciences, Cardiff University, Cardiff, UK; 327 Department of Epidemiology and Biostatistics, Wollo University, Dessie, Ethiopia; 328 Department of Research, Performance Monitoring and Accountability 2020-Ethiopia, Addis Ababa, Ethiopia; 329 Department of Public Health, Arba Minch University, Arba Minch, Ethiopia; 330 Eye Center, Wuhan University, Wuhan, China; 331 University Centre Varazdin, University North, Varazdin, Croatia; 332 International Ph.D. Program in Medicine, Taipei Medical University, Taipei, Taiwan; 333 Research Center for Artificial Intelligence in Medicine, Taipei Medical University, Taipei, Taiwan; 334 National Data Management Center for Health, Ethiopian Public Health Institute, Addis Ababa, Ethiopia; 335 Department of Biomedical Sciences, Mercer University School of Medicine, Macon, GA USA; 336 Department of Surgical Oncology, All India Institute of Medical Sciences, Jodhpur, India; 337 Molecular Biology Unit, Sirius Training and Research Centre, Khartoum, Sudan; 338 Bio-Statistical and Molecular Biology Department, Sirius Training and Research Centre, Khartoum, Sudan; 339 Department of Clinical Pharmacy and Pharmacy Practice, Ahmadu Bello University, Zaria, Nigeria; 340 School of Pharmaceutical Sciences, Universiti Sains Malaysia (University of Science Malaysia), Penang, Malaysia; 341 Department of Pharmacology, Abadan School of Medical Sciences, Abadan, Iran; 342 Department of Biostatistics, Shiraz University of Medical Sciences, Shiraz, Iran; 343 School of Health & Rehabilitation Sciences, The University of Queensland, Brisbane, QLD Australia; 344 Non-communicable Diseases Research Center, Tehran University of Medical Sciences, Tehran, Iran; 345 School of Medicine, Shahid Beheshti University of Medical Sciences, Tehran, Iran; 346 Department of Medicine, Democritus University of Thrace, Alexandroupolis, Greece; 347 College of Medicine and Public Health, Flinders University, Adelaide, Australia; 348 Department of Engineering, Western Sydney University, Sydney, NSW Australia; 349 Comprehensive Cancer Center, University of Alabama at Birmingham, Birmingham, AL USA; 350 Department of Dental Public Health, King Abdulaziz University, Jeddah, Saudi Arabia; 351 Department of Health Policy and Oral Epidemiology, Harvard University, Boston, MA USA; 352 Independent Consultant, Tehran, Iran; 353 Department of Internal Medicine, Ardabil University of Medical Science, Ardabil, Iran; 354 Department of Medical Laboratory Sciences, Adigrat University, Adigrat, Ethiopia; 355 Division of Cardiology, Massachusetts General Hospital, Boston, MA USA; 356 Department of Medical Engineering, University of South Florida, Tampa, FL USA; 357 Department of Surgery, Danang Family Hospital, Danang, Viet Nam; 358 Department of General Medicine, University of Medicine and Pharmacy at Ho Chi Minh City, Ho Chi Minh City, Viet Nam; 359 International Islamic University Islamabad, Islamabad, Pakistan; 360 School of Pharmacy, University of the Western Cape, Cape Town, South Africa; 361 Department of Psychiatry and Behavioural Neurosciences, McMaster University, Hamilton, ON Canada; 362 Department of Psychiatry, University of Lagos, Lagos, Nigeria; 363 Department of Nursing Science, Bowen University, Iwo, Nigeria; 364 Department of Pharmacotherapy and Pharmaceutical Care, Medical University of Warsaw, Warsaw, Poland; 365 School of Medicine, Western Sydney University, Campbelltown, NSW Australia; 366 Department of Optometry and Vision Science, University of KwaZulu-Natal, KwaZulu-Natal, South Africa; 367 Laboratory of Public Health Indicators Analysis and Health Digitalization, Moscow Institute of Physics and Technology, Dolgoprudny, Russia; 368 Department of Medicine, University of Ibadan, Ibadan, Nigeria; 369 Department of Medicine, University College Hospital, Ibadan, Ibadan, Nigeria; 370 Department of Forensic Medicine and Toxicology, Kasturba Medical College, Mangalore, Mangalore, India; 371 Research Department, Nepal Health Research Council, Kathmandu, Nepal; 372 Research Department, Public Health Research Society Nepal, Kathmandu, Nepal; 373 Department of Ophthalmology, Nottingham University Hospitals, QMC Campus, Nottingham, UK; 374 Division of Ophthalmology & Visual Sciences, University of Nottingham, Nottingham, UK; 375 Yonsei University College of Medicine, Seodaemun-gu, South Korea; 376 Global Health Governance Programme, University of Edinburgh, Edinburgh, UK; 377 School of Dentistry, University of Leeds, Leeds, UK; 378 Department of Genetics, Yale University, New Haven, CT USA; 379 Centre for Primary Health Care and Equity, University of New South Wales, Kensington, Australia; 380 Department of Statistics and Econometrics, Bucharest University of Economic Studies, Bucharest, Romania; 381 School of Medicine, Pham Ngoc Thach University of Medicine, Ho Chi Minh City, Viet Nam; 382 College of Health Sciences, VinUniversity, Hanoi, Viet Nam; 383 Department of Health Sciences, Cihan University Sulaimaniya, Sulaymaniyah, Iraq; 384 Cihan University Sulaimaniya Research Center (CUSRC), Sulaymaniyah, Iraq; 385 Sina Trauma and Surgery Research Center, Tehran University of Medical Sciences, Tehran, Iran; 386 Manipal TATA Medical College, Manipal Academy of Higher Education, Manipal, India; 387 Department of Community Medicine, Employees’ State Insurance Model Hospital, Chennai, India; 388 Department of Radiology, Stanford University, Stanford, CA USA; 389 Department of Community Medicine, Mahatma Gandhi Medical College and Research Institute, Puducherry, India; 390 School of Humanities and Social Sciences, Indian Institute of Technology Mandi, Mandi, India; 391 Social Determinants of Health Research Center, Shahid Beheshti University of Medical Sciences, Tehran, Iran; 392 Department of Community Medicine and Family Medicine, All India Institute of Medical Sciences, Gorakhpur, Gorakhpur, India; 393 Department of Health Behaviour, Environment, and Social Medicine, Gadjah Mada University, Yogyakarta, Indonesia; 394 Department of Primary Care and Public Health, Imperial College London, London, UK; 395 Academic Public Health England, Public Health England, London, UK; 396 Department of Medicine, Tehran University of Medical Sciences, Tehran, Iran; 397 Department Biological Sciences, King Abdulaziz University, Jeddah, Egypt; 398 Department of Protein Research, Research and Academic Institution, Alexandria, Egypt; 399 Department of Labour, Directorate of Factories, Government of West Bengal, Kolkata, India; 400 Department of Biostatistics and Epidemiology, International Institute for Population Sciences, Mumbai, India; 401 Faculty of Medicine, Gonabad University of Medical Sciences, Gonabad, Iran; 402 Infectious Diseases Research Center, Gonabad University of Medical Sciences, gonabad, Iran; 403 Sharjah Institute for Medical Research, University of Sharjah, Sharjah, United Arab Emirates; 404 Multidisciplinary Laboratory Foundation University School of Health Sciences (FUSH), Foundation University, Islamabad, Pakistan; 405 International Center of Medical Sciences Research (ICMSR), Islamabad, Pakistan; 406 Ophthalmic Epidemiology Research Center, Shahid Beheshti University of Medical Sciences, Tehran, Iran; 407 Ophthalmic Research Center, Shahid Beheshti University of Medical Sciences, Tehran, Iran; 408 Faculty of Medicine, Bioscience and Nursing, MAHSA University, Selangor, Malaysia; 409 Interdisciplinary Research Centre in Biomedical Materials (IRCBM), COMSATS Institute of Information Technology, Lahore, Pakistan; 410 Research Center for Immunodeficiencies, Tehran University of Medical Sciences, Tehran, Iran; 411 Sharjah Institute of Medical Sciences, University of Sharjah, Sharjah, United Arab Emirates; 412 Applied Biomedical Research Center, Mashhad University of Medical Sciences, Mashhad, Iran; 413 Biotechnology Research Center, Mashhad University of Medical Sciences, Mashhad, Iran; 414 Multiple Sclerosis Research Center, Tehran University of Medical Sciences, Tehran, Iran; 415 Ludwig Maximilian University of Munich, Munich, Germany; 416 Institute for Employment Research, Nuremberg, Germany; 417 College of Medicine, University of Sharjah, Sharjah, United Arab Emirates; 418 Faculty of Pharmacy, Mansoura University, Mansoura, Egypt; 419 Department of Neurology, Charité Universitätsmedizin Berlin (Charité Medical University Berlin), Berlin, Germany; 420 Department of Neurology, University of Southern Denmark, Odense, Denmark; 421 School of Public Health, Taipei Medical University, Taipei, Taiwan; 422 Department of Anatomy, Ras Al Khaimah Medical and Health Sciences University, Ras Al Khaimah, United Arab Emirates; 423 Department of Entomology, Ain Shams University, Cairo, Egypt; 424 Medical Ain Shams Research Institute (MASRI), Ain Shams University, Cairo, Egypt; 425 Department of Pharmacology and Research, All India Institute of Medical Sciences, Jodhpur, India; 426 Indira Gandhi Medical College and Research Institute, Puducherry, India; 427 Faculty of Dentistry, AIMST University, Bedong, Malaysia; 428 Faculty of Medicine, Tehran University of Medical Sciences, Tehran, Iran; 429 Department of Human Genetics and Molecular Medicine, Central University of Punjab, Bathinda, India; 430 Department of Medicine and Surgery, Government Doon Medical College, Dehradun, India; 431 Endocrinology and Metabolism Research Center (EMRC), Tehran University of Medical Sciences, Tehran, Iran; 432 National Heart, Lung, and Blood Institute, National Institute of Health, Rockville, MD USA; 433 Public Health Division, An-Najah National University, Nablus, Palestine; 434 Institute of Molecular Biology and Biotechnology (IMBB), The University of Lahore, Lahore, Pakistan; 435 Research Centre for Health Sciences (RCHS), The University of Lahore, Lahore, Pakistan; 436 Department of Clinical Sciences, Al-Quds University, Ajman, United Arab Emirates; 437 Independent Consultant, Karachi, Pakistan; 438 Department of Pathology and Laboratory Medicine, Northwell Health, New York City, NY USA; 439 Amity Institute of Public Health, Amity University, Noida, India; 440 Department of Ophthalmology, Harvard Medical School, Boston, MA USA; 441 Ophthalmic Research Center (ORC), Shahid Beheshti University of Medical Sciences, Tehran, Iran; 442 Department of Veterinary Public Health and Preventive Medicine, Usmanu Danfodiyo University, Sokoto, Sokoto, Nigeria; 443 Center for Environmental and Respiratory Health Research, University of Oulu, Oulu, Finland; 444 National Institute of Environmental Health Sciences, National Health Research Institutes, Miaoli, Taiwan; 445 Department of Public Health Dentistry, Krishna Vishwa Vidyapeeth (Deemed to be University), Karad, India; 446 Department of Medical-Surgical Nursing, Mazandaran University of Medical Sciences, Sari, Iran; 447 Department of Nursing and Health Sciences, Flinders University, Adelaide, SA Australia; 448 Department of Pediatrics and Child Health Nursing, Dilla University, Dilla, Ethiopia; 449 Unit of Basic Medical Sciences, University of Khartoum, Khartoum, Sudan; 450 Department of Medical Microbiology and Infectious Diseases, Erasmus University, Rotterdam, Netherlands; 451 Family, Health Promotion, and Life Course Department, Pan American Health Organization, Bogota, Colombia; 452 School of Medicine, University of Alabama at Birmingham, Birmingham, AL USA; 453 Department of Medicine Service, US Department of Veterans Affairs (VA), Birmingham, AL USA; 454 Department of Radiodiagnosis, All India Institute of Medical Sciences, Bathinda, India; 455 Department of Ophthalmology, Hywel Dda University Health Board, Llanelli, UK; 456 Department of Nursing, Dire Dawa University, Dire Dawa, Ethiopia; 457 Directive Board, Associação de Profissionais Licenciados de Optometria (Association of Licensed Optometry Professionals), Linda-a-Velha, Portugal; 458 Division of Community Medicine, International Medical University, Kuala Lumpur, Malaysia; 459 Federal Research Institute for Health Organization and Informatics of the Ministry of Health (FRIHOI), Moscow, Russia; 460 Soonchunhyang University, Vision Research Foundation, Cheonan-si, South Korea; 461 Nursing Professional Education Study Program, University Halu Oleo, Kendari, Indonesia; 462 Department of Medical Informatics, Mashhad University of Medical Sciences, Mashhad, Iran; 463 Clinial Research Development Unit, Mashhad University of Medical Sciences, Mashhad, Iran; 464 Department of Clinical Pharmacy, Mekelle University, Mekelle, Ethiopia; 465 Pediatric Intensive Care Unit, King Saud University, Riyadh, Saudi Arabia; 466 Outpatient Department, Wollega University, Bedele town, Ethiopia; 467 Department of Public Health, Wollega University, Nekemte, Ethiopia; 468 Faculty of Public Health, Universitas Sam Ratulangi, Manado, Indonesia; 469 Department of Public Health, Arba Minch College of Health Sciences, Arba Minch, Ethiopia; 470 Department of Medicine, University of Crete, Heraklion, Greece; 471 Department of Nursing, Aksum University, Aksum, Ethiopia; 472 Department of Physiology, East Carolina University, Greenville, NC USA; 473 Department of Epidemiology and Biostatistics, Haramaya University, Harar, Ethiopia; 474 Department of Pharmacology, All India Institute of Medical Sciences, Deoghar, India; 475 Department of Public Health, East Carolina University, Greenville, NC USA; 476 College of Public Health, Temple University, Philadelphia, PA USA; 477 Medical Genomics Research Department, King Abdullah International Medical Research Center, Riyadh, Saudi Arabia; 478 Division of Surgery and Interventional Science, University College London, London, UK; 479 Urmia University of Medical Sciences, Urmia, Iran; 480 Department of Cardiovascular Sciences, Katholieke Universiteit Leuven (University of Leuven), Leuven, Belgium; 481 Department of Public Health, Debre Markos University, Debre Markos, Ethiopia; 482 Department of Ophthalmology Research, Queen Mamohato Memorial Hospital, Maseru, Lesotho; 483 Ophthalmology Unit, Bahir Dar University, Bahirdar, Ethiopia; 484 School of Public Health, Zhejiang University, Zhejiang, China; 485 Department of Public Health Science, Fred Hutchinson Cancer Research Center, Seattle, WA USA; 486 China Center for Health Development Studies, Peking University, Beijing, China; 487 Center for the Study of Aging and Human Development, Duke University, Durham, NC USA; 488 The Russell H. Morgan Department of Radiology and Radiological Science, Johns Hopkins University, Baltimore, MD USA; 489 Department of Health Management, Süleyman Demirel Üniversitesi (Süleyman Demirel University), Isparta, Türkiye; 490 Department of Pharmacology, Bahir Dar University, Bahir Dar, Ethiopia; 491 Pharmacy Department, Alkan Health Science, Business and Technology College, Bahir Dar, Ethiopia; 492 Department of Pediatrics, Kyung Hee University, Seoul, South Korea; 493 Department of Neuropsychopharmacology, National Center of Neurology and Psychiatry, Kodaira, Japan; 494 Department of Public Health, Juntendo University, Tokyo, Japan; 495 Department of Epidemiology and Biostatistics, Wuhan University, Wuhan, China; 496 Department of Bioengineering and Therapeutic Sciences, University of California San Francisco, San Francisco, CA USA; 497 Addictology Department, Russian Medical Academy of Continuous Professional Education, Moscow, Russia; 498 College of Traditional Chinese Medicine, Hebei University, Baoding, China; 499 Department of Ophthalmology, Iran University of Medical Sciences, Tehran, Iran; 500 Department of Biochemistry and Pharmacogenomics, Medical University of Warsaw, Warsaw, Poland; 501 Department of Anatomy, Addis Ababa University, Addis Ababa, Ethiopia; 502 Department of Nursing, Yasuj University of Medical Sciences, Yasuj, Iran

**Keywords:** Epidemiology, Lens diseases

## Abstract

**Background:**

To estimate global and regional trends from 2000 to 2020 of the number of persons visually impaired by cataract and their proportion of the total number of vision-impaired individuals.

**Methods:**

A systematic review and meta-analysis of published population studies and gray literature from 2000 to 2020 was carried out to estimate global and regional trends. We developed prevalence estimates based on modeled distance visual impairment and blindness due to cataract, producing location-, year-, age-, and sex-specific estimates of moderate to severe vision impairment (MSVI presenting visual acuity <6/18, ≥3/60) and blindness (presenting visual acuity <3/60). Estimates are age-standardized using the GBD standard population.

**Results:**

In 2020, among overall (all ages) 43.3 million blind and 295 million with MSVI, 17.0 million (39.6%) people were blind and 83.5 million (28.3%) had MSVI due to cataract blind 60% female, MSVI 59% female. From 1990 to 2020, the count of persons blind (MSVI) due to cataract increased by 29.7%(93.1%) whereas the age-standardized global prevalence of cataract-related blindness improved by −27.5% and MSVI increased by 7.2%. The contribution of cataract to the age-standardized prevalence of blindness exceeded the global figure only in South Asia (62.9%) and Southeast Asia and Oceania (47.9%).

**Conclusions:**

The number of people blind and with MSVI due to cataract has risen over the past 30 years, despite a decrease in the age-standardized prevalence of cataract. This indicates that cataract treatment programs have been beneficial, but population growth and aging have outpaced their impact. Growing numbers of cataract blind indicate that more, better-directed, resources are needed to increase global capacity for cataract surgery.

## Introduction

For 2020, the Global Burden of Disease (GBD) Study reported that cataract remained the leading cause of blindness, with approximately 15.2 million cases [95% Uncertainty Interval (UI): 12.7–18.0) that comprised 45% of global blindness [1]. Cataract also remained the second leading cause of moderate and severe vision impairment (MSVI), with 78.8 million (95% UI: 67.2–91.4) people, which comprised 39% of global MSVI. In spite of global advocacy efforts, such as the VISION 2020 Right to Sight program undertaken by the World Health Organization and International Agency of the Prevention Blindness, and an increase in cataract surgical rates (the number of cataract surgeries per million population) reported across the world, the progress made against cataract has revealed substantial inequality and inequity, with lower-to-middle income countries (LMICs) shouldering the greater burden and having poorer visual outcomes than high-income countries [2–4].

The majority of cataracts are age-related nuclear cataracts, which typically cause vision loss in the sixth decade or later [5–7]. Cataracts, part of the spectrum of diabetic eye disease, are also increasing due to a global epidemic of diabetes, with diabetics more likely to develop cataracts and more quickly lose their vision to cataract compared to people without diabetes [5, 8, 9].

Surgery is the only treatment for cataract, during which an artificial intraocular lens replaces the damaged lens. Cataract surgery is extremely efficacious in terms of restoring sight, and multiple studies have demonstrated its cost-effectiveness, which appears to increase over time [10–12]. In 2015, the International Council of Ophthalmology estimated that globally, there were 14 ophthalmologists performing cataract surgery per million population, but that ranges from less than 1 cataract surgeon per million in low-income countries to as high as 32 in high-income countries, further revealing the global inequity in access to eye care [13]. Age-related cataract exposes another persistent inequity in universal eye health coverage–– men are 1.7 times more likely to undergo cataract surgery than women, and even in high-income countries, women are more likely to wait longer for surgery and experience poorer outcomes [14]. This gender inequity is partially due to the fact that women live longer than men, although sociocultural barriers are also at play. Based on the 2015 GBD Vision Loss Expert Group data, if women had the same access to cataract surgery as men, the blindness burden of cataract could decrease by 11%. However, women, in fact, need more access to surgery than men, to address the gender inequity [14]. Gender differences in cataract burden for 2020 have yet to be analyzed.

With the publication of 2020 GBD vision loss data, there is a need to explore further the global and regional trends in cataract burden since 1990 and better understand the regional and gender inequities of cataract burden. The objective of this article is to provide updated estimates of the global burden of vision loss due to cataract, disaggregated by sex and region, for the period from 2000 to 2020 covered by Global Vision 2020. This is done using the best available ophthalmic epidemiological database, the Global Vision Database which is a comprehensive, continuously updated, online database of ophthalmic epidemiological data curated by the Vision Loss Expert Group (VLEG) [15–17]. Additionally, we assess progress against the goals set out in ‘Towards universal eye health: global action plan 2014–2019 of the World 60 Health Assembly (2013) [18]. This Global Action Plan set a target to reduce the prevalence of avoidable blindness by 25% from 2010 to 2019.

## Methods

The VLEG have maintained, and progressively updated a systematic review of population-based studies of vision impairment and blindness published between Jan 1, 1980, and Oct 1, 2018, including gray literature sources. Data from this systematic review were combined with data from the repository of Rapid Assessment of Avoidable Blindness (RAAB) studies, and data contributed by the GBD obtained from the US National Health and Nutrition Examination survey and the WHO Study on Global Ageing and Adult Health. Detailed methods are published elsewhere [17, 19], and briefly described herein.

In total, the systematic review identified 137 studies, and the VLEG extracted data from 70 studies in 2010, and a further 67 studies in 2014–18 [16]. Studies were primarily national and subnational cross-sectional surveys. The VLEG commissioned the preparation of 5-year age-disaggregated data from the RAAB repository [20]. Studies were included if they met these criteria: population-representative and visual acuity measured using a test chart that could be mapped to Snellen fractions. Studies using self-reported vision loss were excluded. We used the International Classification of Diseases 11th edition criteria for vision loss, as recommended by the WHO, which categorizes people according to presenting better-eye visual acuity. The classification defines moderate vision loss as better eye visual acuity of 6/60 or better but worse than 6/18, severe vision loss as a visual acuity of 3/60 or better but worse than 6/60, and blindness as visual acuity of worse than 3/60 or less than 10° visual field around central fixation.

Data were stratified into datasets including so-called vision-loss envelopes (as per Flaxman et al. [16]) for all-cause mild, moderate, and severe vision loss, and blindness. Data were input into a mixed-effects meta-regression tool developed by the Institute for Health Metrics and Evaluation (IHME) called MR-BRT (meta regression; Bayesian; regularized; trimmed) [21]. Presenting vision impairment defined each level of severity. Prevalence data for under-corrected refractive error were extracted where available, and otherwise calculated by subtracting best-corrected vision impairment from presenting vision impairment for each level of severity in studies that reported both measures for a given location, sex, age group, and year. Other causes were quantified as part of the best-corrected estimates of vision impairment at each level of severity. Minimum age for inclusion of data was defined as 20 years for cataract.

We generated location, year, age, and sex-specific estimates of MSVI and blindness using Disease Modeling Meta-Regression (Dismod-MR) 2.1; [19] the data processing steps are described elsewhere [17]. In brief, Dismod-MR 2.1 models were run for all vision impairment strata (moderate, severe, blindness) regardless of cause and, separately, for MSVI and blindness for each modeled cause of vision impairment. Then, models of MSVI due to cataract were split into moderate and severe estimates using the ratio of overall prevalence in the all-cause moderate presenting vision impairment and severe presenting vision impairment models. Next, prevalence estimates for cataract were stratified by severity were scaled to the models of all-cause prevalence by severity. This produced final estimates by age, sex, year, and location for cataract vision impairment stratified by severity. We age-standardized our estimates using the GBD standard population [22]. Data on blindness and MSVI due to AMD were presented by seven super-regions (Southeast Asia/East Asia/Oceania, Central Europe/Eastern Europe/Central Asia, High-income, Latin America and Caribbean, North Africa and Middle East, South Asia, and Sub-Saharan Africa) and globally. Data on other causes of vision impairment and blindness will be presented in separate publications.

The funder of the study had no role in study design, data collection, data analysis, data interpretation, or writing of the report. The corresponding author had full access to all the data in the study and had final responsibility for the decision to submit for publication.

## Results

In 2020, 17.01 million (all ages, 95% uncertainty interval (UI) 14.40–19.93) people were blind due to cataract (Table 1). This breaks down by gender as 6.78 million (95% UI 5.73–7.98) men and 10.22 million (95% UI 8.76–11.96) women blind from cataract (Table 2). The majority of these are over 50 years of age with 15.17 million (95% UI 12.70–18.00) so affected (Table 1). Of these, 5.96 million (95% UI 4.98–7.11) men and 9.22 million (95% UI 7.73–10.88) women are blind from cataract (Table 2).

**Table 1:** Number of people of all ages (mean [95% UI]) with blindness (presenting visual acuity <3/60) or MSVI (presenting visual acuity <6/18, ≥3/60) due to Cataract, the age-standardized prevalence (%) in people aged ≥50 years (mean [95% UI]), and the percentage of all blindness or MSVI attributed to Cataract in people aged ≥50 years (95% UI) in world regions in 2020.

		Blindness due to Cataract in 2020			MSVI due to Cataract in 2020		
World Region	2020, total population (‘000 s)	Number of people (‘000 s) with blindness in 2020 (all ages)	Age-standardized prevalence of Cataract blindness in 2020 (aged ≥50 years)	Percentage of all Cataract blindness in 2020 (≥50 years)	Number of people (‘000 s) with MSVI in 2020 (all ages)	Age-standardized prevalence of Cataract MSVI in people ≥50 years aged in 2020	Percentage of Cataract MSVI in 2020 (≥50 years)
Global	7,890,000	17,005 (14,395–19,926)	0.84 (0.70–0.99)	39.55 (33.48–46.34)	83,475(71,758–95,983)	4.34 (3.71–5.02)	28.30 (24.32–32.54)
Southeast Asia, East Asia, and Oceania	2,192,710	6305 (5322–7445)	0.97 (0.81–1.15)	41.82 (35.30–49.38)	28,238 (24,354–32,390)	4.71 (4.04–5.41)	34.00 (29.32–39.00)
Central Europe, Eastern Europe, and Central Asia	417,291	291 (234–356)	0.19 (0.15–0.23)	20.53 (16.50–25.15)	3172(2620–3733)	2.13 (1.75–2.52)	17.64 (14.57–20.76)
High-income	1,087,856	505(410–619)	0.09 (0.07–0.11)	16.82 (13.66–20.60)	8214 (6955–9574)	1.46 (1.22–1.71)	26.44 (22.39–30.82)
Latin America and Caribbean	601,551	1101 (910–1320)	0.78 (0.64–0.95)	30.11 (24.90–36.10)	4658 (3935–5391)	3.39 (2.85–3.96)	19.03 (16.07–22.02)
North Africa and Middle East	631,727	844 (676–1037)	0.91 (0.71–1.14)	27.31 (21.88–33.57)	5399 (4573–6303)	5.81 (4.92–6.80)	24.73 (20.94–28.87)
South Asia	1,841,435	6352 (5373–74169)	2.23 (1.89–2.61)	53.20 (45.00–62.11)	28,744 (24,674–33,511)	9.46 (8.11–10.93)	29.87 (25.64–34.83)
Sub-Saharan Africa	1,114,806	1604 (1358–1857)	1.49 (1.24–1.78)	31.56 (26.71–36.53)	5047 (4342–5781)	5.14 (4.40–5.93)	24.69 (21.24–28.28)

**Table 2:** Number of males and females with blindness (presenting visual acuity <3/60), and the age-standardized prevalence (% [95% UI]) of blindness due to Cataract (all ages and people aged ≥50 years) in 2020.

	Total Population	Total number of Cataract blindness and aged-standardized Cataract blindness in 2020 (all ages)				Total number of Cataract blindness and aged-standardized Cataract blindness in people aged 50+ years in 2020			
World Region	2020, total population (‘000 s)	Number of males(‘000 s) with Cataract blindness in 2020	Number of females (‘000 s) with Cataract blindness in 2020	Age standardized prevalence of blindness in males in 2020	Age standardized prevalence of blindness in females in 2020	Number of males(‘000 s) (50+ years) with Cataract blindness in 2020	Number of females(‘000 s) (50+ years) with Cataract blindness in 2020	Age standardized prevalence of blindness in males in 2020	Age standardized prevalence of blindness in females in 2020
Global	7,890,000	6781 (5733–7978)	10,224 (8671–11,956)	0.18 (0.15–0.21)	0.23 (0.19–0.27)	5957 (4978–7110)	9217 (7728–10,884)	0.73 (0.61–0.87)	0.93 (0.78–1.09)
Southeast Asia, East Asia, and Oceania	2,192,710	2297 (1929–2722)	4007 (3394–4714)	0.18 (0.16–0.22)	0.29 (0.24–0.34)	1966 (1626–2360)	3577 (2990–4235)	0.73 (0.61–0.88)	1.16 (0.97–1.38)
Central Europe, Eastern Europe, and Central Asia	417,291	102(81–124)	188 (152–232)	0.04 (0.04–0.05)	0.05 (0.04–0.06)	88 (70–111)	177 (142–220)	0.17 (0.14–0.22)	0.20 (0.16–0.24)
High-income	1,087,856	221(178–267)	284 (231–351)	0.02 (0.02–0.03)	0.02 (0.02–0.03)	196 (157–241)	260 (209–325)	0.09 (0.07–0.11)	0.08 (0.07–0.11)
Latin America and Caribbean	601,551	483 (397–579)	617 (512–738)	0.18 (0.15–0.22)	0.19 (0.16–0.23)	433 (353–529)	571 (469–691)	0.76 (0.62–0.93)	0.80 (0.66–0.97)
North Africa and Middle East	631,727	314 (249–389)	529 (424–648)	0.16 (0.13–0.20)	0.26 (0.21–0.32)	276 (214–350)	478 (378–595)	0.69 (0.54–0.86)	1.11 (0.88–1.37)
South Asia	1,841,435	2706 (2293–3166)	3646 (3087–4262)	0.45 (0.38–0.52)	0.57 (0.48–0.66)	2497 (2096–2939)	3414 (2883–4026)	1.95 (1.65–2.27)	2.48 (2.10–2.90)
Sub-Saharan Africa	1,114,806	655 (555–762)	949 (801–1097)	0.33 (0.27–0.38)	0.40 (0.34–0.47)	498 (415–605)	737 (615–885)	1.32 (1.09–1.59)	1.63 (1.36–1.94)

Overall, 83.48 million (95% UI 71.76–96.98) people are estimated to have MSVI from cataract (Table 1). Of these 34.59 million (95% UI 29.69–39.95) are men, and 48.89 million (95% UI 42.05–56.06) are women (Table 3). Again, the majority are over 50 years of age, 78.79 million (95% UI 67.20–91.40) people, 32.41 million (95% UI 27.55–37.74) men and 46.38 million (95% UI 39.66–53.66) women suffer from MSVI due to cataract (Tables 1 and 3).

**Table 3:** Number of males and females with Cataract MSVI, and the age-standardized prevalence (% [95% uncertainty intervals (UIs)]) of Cataract MSVI (all ages and people aged ≥50 years) in 2020.

	Total population	Total number of Cataract MSVI and aged-standardized Cataract MSVI in 2020 (all ages)				Total number of Cataract MSVI and aged-standardized Cataract MSVI in people aged 50+ years in 2020			
World Region	2020, total population (‘000 s)	Number of males(‘000 s) with Cataract MSVI in 2020	Number of females (‘000 s) with Cataract MSVI in 2020	Age standardized prevalence of MSVI in males in 2020	Age standardized prevalence of MSVI in females in 2020	Number of males(‘000 s) (50+ years) with Cataract MSVI in 2020	Number of females(‘000 s) (50+ years) with Cataract MSVI in 2020	Age standardized prevalence of MSVI in males in 2020	Age standardized prevalence of MSVI in females in 2020
Global	7,890,000	34,585 (29,694–39,953)	48,889 (42,051–56,064)	0.92 (0.79–1.05)	1.08 (0.93–1.24)	32,410 (27,550–37,744)	46,375 (39,663–53,662)	3.96 (3.38–4.59)	4.67 (3.99–5.40)
Southeast Asia, East Asia, and Oceania	2,192,710	11,351(9749–13,032)	16,887 (14,577–19,432)	0.94 (0.81–1.07)	1.20 (1.04–1.37)	10,676 (9,096–12,387)	16,164 (13,880–18,651)	4.06 (3.47–4.67)	5.24 (4.51–6.00)
Central Europe, Eastern Europe, and Central Asia	417,291	978 (805–1157)	2194 (1806–2599)	0.42 (0.35–0.49)	0.53 (0.44–0.63)	922(750–1101)	2123 (1740–2524)	1.80 (1.48–2.13)	2.31 (1.90–2.74)
High-income	1,087,856	2985(2525–3485)	5228 (4431–6107)	0.31 (0.26–0.36)	0.38 (0.32–0.43)	2811 (2349–3306)	5071 (4278–5945)	1.27 (1.06–1.48)	1.59 (1.34–1.87)
Latin America and Caribbean	601,551	2057 (1739–2390)	2600 (2202–3008)	0.78 (0.66–0.91)	0.79 (0.67–0.92)	1915 (1599–2242)	2434 (2045–2847)	3.38 (2.83–3.95)	3.40 (2.86–3.99)
North Africa and Middle East	631,727	2405 (2034–2811)	2993 (2539–3482)	1.22 (1.03–1.43)	1.43 (1.22–1.67)	2229(1868–2644)	2795 (2354–3288)	5.35 (4.52–6.30)	6.27 (5.29–7.32)
South Asia	1,841,435	12,732 (10,878–14,943)	16,011 (13,766–18,590)	1.98 (1.71–2.30)	2.32 (2.00–2.67)	12,017 (10,208–14,116)	15,187 (12,989–17,698)	8.71 (7.46–10.12)	10.19 (8.74–11.74)
Sub-Saharan Africa	1,1148,06	2073 (1780–2398)	2973 (2565–3392)	1.10 (0.95–1.27)	1.29 (1.11–1.47)	1837 (1562–2147)	2599 (2219–3020)	4.76 (4.08–5.52)	5.47 (4.69–6.29)

Cataract caused 39.55% (95% UI: 33.48, 46.34%) of all blindness in 2020 worldwide. Regionally, the highest proportion of cataract-related blindness was found in South Asia (53.20 [95% UI: 45.00, 62.11%]) and Southeast Asia, East Asia, and Oceania (41.82% [95% UI: 35.30, 49.38]) (Table 1). The regions with the lowest proportion of all cataract-related blindness of all blind individuals were High Income Countries (16.82% [UI: 13.66, 20.60]), and Central Europe, Eastern Europe, and Central Asia (20.53% [95% UI: 16.50, 25.15]). Cataract caused 28.30% (95% UI: 24.32, 32.54) of all cases with MSVI in 2020 worldwide. Southeast Asia, East Asia, and Oceania (34.00 (29.32-39.00)% [95% UI: 29.32, 39.00]), and South Asia (29.87% [95% UI: 25.64, 34.83]) were regions with the highest percentage of cataract-related MSVI of all visually impaired individuals (Table 1).

**Fig. 1:**
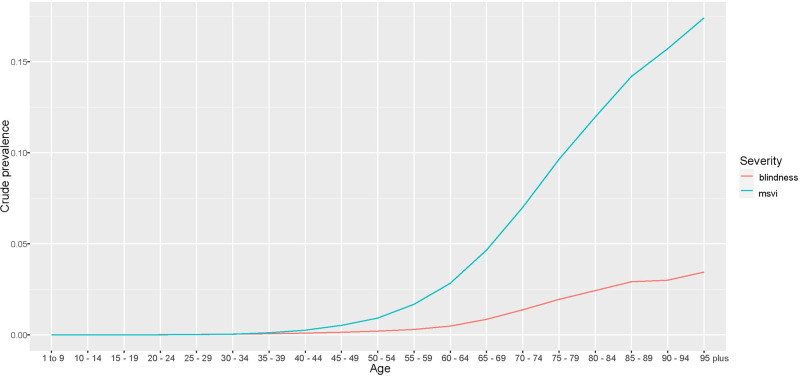
Crude prevalence of Blindness and MSVI due to cataract in 2020 globally by age.

In 2020, the global age-standardized prevalence of cataract-related blindness in those aged ≥50 years was 0.84% (95% UI: 0.70, 0.99) and for cataract-related MSVI was 1.01% (95% UI: 0.87, 1.15) (Table 1). The variation of crude prevalence with age is shown in Fig. 1. The regions with the highest age-standardized prevalence of cataract-related blindness were South Asia (2.23% [95% UI: 1.89, 2.61]) and Sub-Saharan Africa (1.49% [95% UI: 1.24, 1.78]). The lowest age-standardized prevalence of cataract blindness in 2020 was in the regions of High Income Countries (0.09% [95% UI: 0.07, 0.11]) and Central Europe, Eastern Europe, and Central Asia (0.19% [95% UI: 0.15, 0.23]). The regions with the highest age-standardized prevalence of cataract-related MSVI in 2020 were South Asia (2.15% [95% UI: 1.85, 2.49]), and North Africa and the Middle East (1.33% [95% UI: 1.13, 1.55]). The lowest figures were found in high-income countries (0.35% [95% UI: 0.30, 0.40]) and Central Europe, Eastern Europe, and Central Asia (0.49% [95% UI: 0.41, 0.58]) (Table 1). The variation in these results by gender across the regions are shown in Tables 2 and 3.

Between 2000 and 2020, the global percentage change in age-standardized prevalence of cataract-related blindness among adults ≥50 years decreased by 27.54% (95% UI: −27.68, −27.39), among males by −31.78% (95%UI −31.91, −31.64) and by 24.82% in females (95% UI: −24.97, −24.68) (Table 4). However, the absolute number of cases (unadjusted for age) increased by 29.72% (95% UI: 29.46, 29.98), in males 25.65% (95% CI 25.39, 25.92) and in females 32.49% (95% CI: 32.23, 32.75). An especially large reduction in the age-standardized prevalence of cataract-related blindness amongst adults aged ≥50 years (both sexes) was found in Southeast Asia, East Asia and Oceania (−42.99% [95% UI: −43.10, −42.88]), North Africa and Middle East (−39.97% [95% UI: −40.13, −39.81]) and South Asia (−36.53% [95% UI: −36.65, −36.41]), with a modest reduction in high-income countries (−6.86% [95% UI: −7.10, −6.62]) (Table 4). The greatest percentage increases in absolute number of cases were in Latin America and the Caribbean 71.25% (95% UI 70.86, 71.64) and in high income countries 49.30 (95% UI 48.92, 49.69). Only Central Europe, Eastern Europe, and Central Asia showed a reduction in the caseload (−4.40% [95% UI −4.66, −4.14].

**Table 4:** Percentage change in crude prevalence of Cataract blindness (presenting visual acuity <3/60) in adults age 50 years and older between 2000 and 2020 by world super region.

	Crude Prevalence			Number of Cases (‘000 s)			Age standardized prevalence		
World Region	Male (%, 95% UI)	Female (%, 95% UI)	Both (%, 95% UI)	Male (%, 95% UI)	Female (%, 95% UI)	Both (%, 95% UI)	Male (%, 95% UI)	Female (%, 95% UI)	Both (%, 95% UI)
Global	−28.75 (−28.90−28.61)	−24.58 (−24.73−24.43)	−26.30 (−26.45−26.15)	25.65 (25.39–25.92)	32.49 (32.23–32.75)	29.72 (29.46–29.98)	−31.78 (−31.91−31.64)	−24.82 (−24.97−24.68)	−27.54 (−27.68−27.39)
High-income	7.88 (7.61–8.16)	2.58 (2.30–2.85)	4.51 (4.24–4.78)	58.76 (58.36–59.16)	42.88 (42.50–43.26)	49.30 (48.92–49.69)	−6.69 (−6.93−6.46)	−7.16 (−7.40−6.91)	−6.86 (−7.10−6.62)
Central Europe, Eastern Europe, and Central Asia	−19.82 (−20.04−19.60)	−25.74 (−25.94−25.55)	−24.23 (−24.44−24.02)	4.00 (3.72–4.29)	−8.13 (−8.38−7.89)	−4.40 (−4.66−4.14)	−26.60 (−26.79−26.41)	−31.03 (−31.21−30.85)	−30.14 (−30.33−29.96)
Latin America and Caribbean	−19.70 (−19.89−19.51)	−10.77 (−10.97−10.57)	−14.83 (−15.02−14.64)	58.24 (57.87–58.62)	82.64 (82.23–83.05)	71.25 (70.86–71.64)	−23.99 (−24.16−23.81)	−18.39 (−18.57−18.20)	−20.83 (−21.01−20.66)
North Africa and Middle East	−45.07 (−45.23−44.92)	−39.04 (−39.20−38.88)	−41.39 (−41.55−41.23)	14.12 (13.80–14.44)	26.84 (26.51–27.18)	21.87 (21.54–22.19)	−41.99 (−42.15−41.83)	−39.64 (−39.80−39.49)	−39.97 (−40.13−39.81)
South Asia	−30.68 (−30.81−30.54)	−32.04 (−32.17−31.90)	−31.10 (−31.23−30.96)	30.83 (30.57–31.09)	37.40 (37.13–37.67)	34.55 (34.28–34.81)	−34.95 (−35.06−34.83)	−38.63 (−38.74−38.52)	−36.53 (−36.65−36.41)
Southeast Asia, East Asia, and Oceania	−41.72 (−41.84−41.59)	−38.94 (−39.06−38.82)	−39.73 (−39.85−39.61)	15.24 (14.99–15.49)	24.55 (24.30–24.80)	21.08 (20.83–21.33)	−46.86 (−46.97−46.75)	−40.55 (−40.67−40.44)	−42.99 (−43.10−42.88)
Sub-Saharan Africa	−29.68 (−29.84−29.52)	−28.83 (−28.98−28.68)	−28.74 (−28.90−28.59)	24.41 (24.13–24.68)	38.07 (37.77–38.36)	32.21 (31.92–32.50)	−25.87 (−26.03−25.71)	−24.19 (−24.34−24.03)	−24.93 (−25.08−24.77)

Between 2000 and 2020, the global percentage change in age-standardized prevalence of cataract MSVI among adults ( ≥ 50 years) increased (7.17% [95% UI: 6.98, 7.36]), among males (4.70% [95% UI 4.52, 4.89]) and females (8.94% [95% UI: 8.75, 9.13]) (Table 5). However, the absolute number of cases increased by 93.11% (95% UI: 92.75, 93.46), in males 93.69% (95% CI 93.32, 94.05) and in females 92.70% (95% CI: 92.36, 93.04). Sub-Saharan Africa (2.29% [95%UI 2.12, 2.47]) and Southeast Asia, East Asia and Oceania 1.96% [95%UI 1.78, 2.13]) were the only world regions where a substantial increase in the age-standardized prevalence of cataract MSVI was observed with notable decreases in South Asia (-5.53 [95% UI: -5.69, -5.37]) and Latin America and Caribbean (-4.83% [95% UI: -5.01, -4.65]). The increase in the absolute number of cataract MSVI cases was greatest in Southeast Asia, East Asia, and Oceania (115.21% [95%UI 114.83, 115.58]), and least in Central Europe, Eastern Europe, and Central Asia (38.18% [95%UI 37.87, 38.49]) (Table 5).

**Table 5:** Percentage change in crude prevalence of Cataract MSVI (presenting visual acuity <6/18, ≥3/60) and in adults aged 50 years and older between 2000 and 2020 by world super region.

	Crude prevalence			Number of cases (‘000 s)			Age standardized prevalence		
World Region	Male (%, 95% UI)	Female (%, 95% UI)	Both (%, 95% UI)	Male (%, 95% UI)	Female (%, 95% UI)	Both (%, 95% UI)	Male (%, 95% UI)	Female (%, 95% UI)	Both (%, 95% UI)
Global	9.82 (9.61–10.03)	9.70 (9.50–9.89)	9.72 (9.52–9.92)	93.69 (93.32–94.05)	92.70 (92.36–93.04)	93.11 (92.75–93.46)	4.70 (4.52–4.89)	8.94 (8.75–9.13)	7.17 (6.98–7.36)
High-income	20.98 (20.74–21.23)	9.66 (9.44–9.88)	12.62 (12.40–12.84)	78.04 (77.67–78.40)	52.75 (52.45–53.05)	60.90 (60.58–61.21)	1.56 (1.36–1.76)	0.02 (−0.18–0.21)	−0.48 (−0.67–−0.28)
Central Europe, Eastern Europe, and Central Asia	12.39 (12.13–12.66)	9.21 (8.96–9.46)	9.52 (9.27–9.77)	45.79 (45.45–46.13)	35.12 (34.81–35.42)	38.18 (37.87–38.49)	0.96 (0.74–1.17)	1.33 (1.11–1.55)	0.46 (0.24–0.68)
Latin America and Caribbean	−0.64 (−0.83–−0.44)	2.41 (2.22–2.61)	1.11 (0.91–1.31)	95.81 (95.42–96.19)	109.62 (109.21–110.02)	103.30 (102.91–103.70)	−5.59 (−5.77–−5.41)	−4.22 (−4.40–−4.04)	−4.83 (−5.01–−4.65)
North Africa and Middle East	−6.92 (−7.11–−6.73)	−3.43 (−3.62–−3.24)	−5.00 (−5.19–−4.81)	93.39 (92.99–93.78)	100.96 (100.57–101.35)	97.53 (97.14–97.92)	−1.03 (−1.22–−0.84)	−2.89 (−3.07–−2.71)	−1.92 (−2.10–−1.73)
South Asia	0.83 (0.64–1.02)	−3.41 (−3.59–−3.23)	−1.14 (−1.33–−0.96)	90.29 (89.93–90.65)	95.27 (94.91–95.63)	93.04 (92.68–93.40)	−3.46 (−3.63–−3.29)	−7.64 (−7.80–−7.48)	−5.53 (−5.69–−5.37)
Southeast Asia, East Asia, and Oceania	8.65 (8.45–8.85)	5.62 (5.43–5.80)	7.12 (6.93–7.31)	114.84 (114.44–115.24)	115.45 (115.07–115.83)	115.21 (114.83–115.58)	−0.70 (−0.87–−0.53)	3.53 (3.36–3.70)	1.96 (1.78–2.13)
Sub-Saharan Africa	−6.04 (−6.22–−5.87)	−0.15 (−0.33–0.03)	−2.29 (−2.47–−2.11)	66.22 (65.91–66.53)	93.70 (93.35–94.04)	81.29 (80.96–81.62)	−0.60 (−0.78–−0.43)	4.70 (4.52–4.88)	2.29 (2.12–2.47)

## Discussion

Cataract, the world’s leading cause of blindness, remains one of the greatest opportunities in global health to make impactful and cost-effective contributions. Cataract surgery is safe and highly effective with both higher and lower technology approaches (e.g., phacoemulsification or extracapsular techniques respectively), and can be provided relatively inexpensively [23]. As a surgical condition, it requires a system able to provide one-at-a-time clinical care, like most causes of blindness and visual impairment. There are various eye service delivery models that can be used to address the cataract burden. However, it makes sense to combine it in a system with other ophthalmic services, ethically addressing other issues that will come to the attention of the service as well as providing a more professional-friendly work environment to retain capable eye care professionals (ophthalmologists, optometrists, eye nurses and others).

As an endemic condition, the ideal approach to the problem is to develop sufficient capacity and health system functionality to make ophthalmic surgery widely available worldwide. Given the relatively low level of infrastructure and consumables required for quality surgery, government health systems are well positioned to address this issue for the economically poorest persons. While funding limitations may constrain their systems’ scale [24], cataract surgery has considerable economic and quality of life benefits compared to its cost [10], which can offset the investment. Moreover, several health systems in different locations have demonstrated that self-sustaining services can be provided at costs most patients are willing to pay while also generating surpluses to provide service to the very poor [25, 26]. Such “cross-subsidizing” systems have made a large contribution to alleviating cataract blindness in much of the world, although these require a dominant service provider e.g. Aravind Eye Care System in South India. Systems for eye care should contemplate the value of ”patient financial contribution” for cataract surgery as much as possible; offering universal free or highly subsidized surgery may unnecessarily leave that health care financing resource at the table. In addition, surgical campaigns have been used extensively to deal with “backlogs” in cataract blindness; these are ideal for unreached/remote areas where development is unlikely to reach the cataract blind on a reasonable time scale without interfering with the ultimate solution of local capacity development. Our data demonstrate that these sorts of efforts have been fruitful in reducing the per capita levels of cataract blindness over the last 20 years over much of the world. Indeed, the World Health Assembly Global Action Plan target of a 25% reduction from 2010 to 2019 in avoidable vision impairment (WHA 66.3 24/5/2013) was met for cataract blindness (from an age-adjusted prevalence perspective) [18].

However, the successes have not kept pace with the impact of population growth and aging, with the result that the number of cataract blind is substantially increasing. Cataract also remains the leading cause of blindness despite these improvements and its favorable treatability. Thus, further investment in sustainable health systems able to provide quality cataract surgeries is likely to provide very substantial societal and economic net benefits. Because development is a long-term proposition, sustained commitment will be needed, whether through committed funders (e.g., government or charity programs) or self-sustaining organizations (private non-profit or social enterprise systems, or government systems allowing cost recovery).

While our data demonstrate a notable improvement in blindness (worse than 20/400 visual acuity), we did not see a similar decrease in MSVI (worse than 20/60 to 20/400) which also is associated with substantial disability/economic impact [27, 28]. Indeed, MSVI became more prevalent and nearly doubled in the number of cases. This pattern suggests successful targeting of the most severely impaired cases, albeit at the neglect of the less severely impaired. However, MSVI also needs to be targeted to alleviate visual disability and its socioeconomic impacts [27, 28]. Indeed, research into willingness to pay for cataract surgery suggests that people in the MSVI range (e.g., younger people otherwise capable of employment) may be more willing to pay for cataract surgery than more severe “blind” persons [26]. Expansion of the indications for cataract surgery may be needed to accomplish improvements in cataract MSVI also [29].

The WHO criteria score blindness and visual impairment based on the vision in the better eye. Following this logic, it would seem sensible in an economically constrained environment to focus on operating one eye. However, second eye surgeries also have important benefits to vision, visual ability and well-being [30, 31], and has been shown to have very high cost-effectiveness (cost per quality-adjusted life year gained) and a favorable cost-effectiveness in an evidence-based review [32, 33]. In addition, second eye surgery provides insurance that vision could continue in the event something happened to the first eye for persons in locations with poor service access. Binocular vision is important for activities requiring depth perception, falls prevention, increases contrast sensitivity and provides better binocular visual acuity than single eye surgery alone [31]. Because case finding of second eye cataracts and second eye operations have less marginal cost for bilateral cases than first eye cataracts [34], it is desirable to operate second eyes as well. Persons also may be more willing to pay for a cataract surgery after seeing the result of first eye cataract surgery [33]. Second eye cataract surgeries generally should be made available to patients in cataract programs, especially if patients are willing to pay some or all of the cost.

While improvements in cataract blindness were observed over the last 20 years, huge disparities in the prevalence remain between low- and high-income regions. South Asia has the highest number of cataract blind and by far the highest prevalence, a significant focus in this super region has the greatest potential for improvement. However, other poor regions (e.g., Sub-Saharan Africa) which are expected to see a growth in the elderly population in coming years and have a very high prevalence of cataract blindness amongst the elderly needing aggressive efforts to develop an eye care system capable of handling the volume of cataract surgery and other eye care services which can be forecast to be needed. Given the very low number of ophthalmologists and other eye care professionals in these areas, the time is now to strengthen and expand both training and systems for eye care delivery [34].

Our results demonstrated again that women are disproportionately represented amongst the cataract blind and visually impaired, and that the inequity is widening. The extent of this difference varies across the globe, but is generally consistent. The difference might reflect differences in family willingness to pay for male and female surgery [35]. Differences in acceptance of surgery between males and females could be another explanation. However, acceptance of clinical services tends to be higher among women than men in high income settings. Notably, female survival is generally longer than male survival which might be associated with a higher burden of age-related cataract even if service utilization were equal. Baruwa et al found that five years’ access to free cataract screening and low-cost high quality cataract surgery was associated with equalization in willingness to pay for cataract surgery across males and females [36]. Improving cataract surgery quality, community knowledge of the benefits of cataract surgery, and reducing barriers to surgical access likely are among the core strategies that need to be implemented in order to overcome the male-female gap in cataract surgery utilization. Without foregoing the promotion of cataract surgery among males, who also need to increase cataract surgery utilization, female surgery promoters and other strategies to increase female use of cataract surgery also could be helpful to reduce the disproportionately higher female cataract blindness and visual impairment burden.

The impact of the COVID-19 pandemic on cataract blindness is unclear at this time. Emerging evidence that service delivery was adversely affected during the emergency phase of the pandemic may drive the cataract burden up [37]. This may be offset by global decreases in life expectance from the disease and its sequelae [37]. These impacts may not be visible for several years, but are likely to be overwhelmed by existing trajectories of population growth and ageing.

In summary, as the population grows and ages while coverage of cataract surgery remains incomplete, immense numbers of people remain blind and vision impaired from cataract. These numbers are expected to continue growing markedly as the population increases and ages worldwide, especially in the least developed countries with young but rapidly aging populations and high cataract blindness/MSVI prevalence. While much has been achieved by initiatives to tackle cataract blindness, much more needs to be done to provide cataract surgery to those in need. Programs for delivering cataract to the vision impaired should not only target the blind, but also those with MSVI who also substantially benefit from treatment and appear to be under-targeted. High quality service provision is essential for inciting demand for cataract surgery, and thus is a key issue along with increasing the number of surgeries. Ophthalmologist training, which takes a long time, needs to be developed urgently in areas of insufficient coverage. Eye care systems in which ophthalmologists can operate successfully and other eye care professionals can work successful also are very important. While all regions with substantial numbers of cataract blind need increased services, females especially need to access cataract surgery more. Culturally appropriate efforts to promote female cataract surgery are an important piece of what needs to be done. Globally, immense increases in resource mobilization for treating cataract are required. All sources of healthcare financing need to be tapped to develop sustainable eye care systems able to tackle the cataract problem with high quality surgery.

## Summary

### What was known before

- Globally, in 2020, 17.0 million people were blind and nearly 83.5 million were visually impaired by cataract.

### What this study adds

- The contribution of cataract to blindness and moderate and severe vision impairment (MSVI) by region and the change in this contribution between 2000 and 2020. The change in global age-standardized prevalence of cataract-related blindness and MSVI between 2000 and 2020 and the differences by sex and region.

### Supplementary information

Supplementary File

## Data Availability

Data sources for the Global Vision Database are listed at the following weblink http://www.anglia.ac.uk/verigbd. Fully disaggregated data is not available publicly due to data sharing agreements with some principal investigators yet requests for summary data can be made to the corresponding author.
