# Publisher Correction: Global estimates on the number of people blind or visually impaired by cataract: a meta-analysis from 2000 to 2020

**DOI:** 10.1038/s41433-024-03161-7

**Published:** 2024-07-16

**Authors:** Vision Loss Expert Group of the Global Burden of Disease Study, Konrad Pesudovs, Van Charles Lansingh, John H. Kempen, Ian Tapply, Arthur G. Fernandes, Maria Vittoria Cicinelli, Alessandro Arrigo, Nicolas Leveziel, Serge Resnikoff, Hugh R. Taylor, Tabassom Sedighi, Seth Flaxman, Mukkharram M. Bikbov, Tasanee Braithwaite, Alain Bron, Ching-Yu Cheng, Monte A. Del Monte, Joshua R. Ehrlich, Leon B. Ellwein, David Friedman, João M. Furtado, Gus Gazzard, Ronnie George, M. Elizabeth Hartnett, Jost B. Jonas, Rim Kahloun, Moncef Khairallah, Rohit C. Khanna, Janet Leasher, Julie-Anne Little, Vinay Nangia, Michal Nowak, Tunde Peto, Pradeep Ramulu, Fotis Topouzis, Mitiadis Tsilimbaris, Ya Xing Wang, Ningli Wang, Rupert R. A. Bourne, GBD 2019 Blindness and Vision Impairment Collaborators, Konrad Pesudovs, Van Charles Lansingh, John H. Kempen, Ian Tapply, Arthur G. Fernandes, Maria Vittoria Cicinelli, Alessandro Arrigo, Nicolas Leveziel, Paul Svitil Briant, Theo Vos, Serge Resnikoff, Hugh R. Taylor, Tabassom Sedighi, Seth Flaxman, Yohannes Habtegiorgis Abate, Mohammad Abdollahi, Mozhan Abdollahi, Ayele Mamo Abebe, Olumide Abiodun, Richard Gyan Aboagye, Woldu Aberhe Abrha, Hasan Abualruz, Hiwa Abubaker Ali, Eman Abu-Gharbieh, Salahdein Aburuz, Tadele Girum Girum Adal, Mesafint Molla Adane, Isaac Yeboah Addo, Qorinah Estiningtyas Sakilah Adnani, Muhammad Sohail Afzal, Shahin Aghamiri, Bright Opoku Ahinkorah, Aqeel Ahmad, Sajjad Ahmad, Ali Ahmadi, Ayman Ahmed, Haroon Ahmed, Ahmad Samir Alfaar, Abid Ali, Syed Shujait Shujait Ali, Awais Altaf, Hubert Amu, Sofia Androudi, Rodrigo Anguita, Abhishek Anil, Saeid Anvari, Anayochukwu Edward Anyasodor, Francis Appiah, Jalal Arabloo, Mosab Arafat, Damelash Areda, Reza Arefnezhad, Brhane Berhe Aregawi, Akeza Awealom Asgedom, Tahira Ashraf, Seyyed Shamsadin Athari, Bantalem Tilaye Tilaye Atinafu, Maha Moh’d Wahbi Atout, Alok Atreya, Haleh Ayatollahi, Ahmed Y. Azzam, Hassan Babamohamadi, Sara Bagherieh, Yogesh Bahurupi, Atif Amin Baig, Biswajit Banik, Mainak Bardhan, Saurav Basu, Kavita Batra, Nebiyou Simegnew Bayileyegn, Fatemeh Bazvand, Addisu Shunu Beyene, Devidas S. Bhagat, Akshaya Srikanth Bhagavathula, Pankaj Bhardwaj, Sonu Bhaskar, Jasvinder Singh Bhatti, Mukharram Bikbov, Niloufar Bineshfar, Marina G. Birck, Veera R. Bitra, Tasanee Braithwaite, Katrin Burkart, Yasser Bustanji, Zahid A. Butt, Florentino Luciano Caetano dos Santos, Luis Alberto Cámera, Vera L. A. Carneiro, Muthia Cenderadewi, Eeshwar K. Chandrasekar, Vijay Kumar Chattu, Nitin Chitranshi, Hitesh Chopra, Dinh-Toi Chu, Kaleb Coberly, João M. Coelho, Natália Cruz-Martins, Omid Dadras, Xiaochen Dai, Subasish Das, Ana Maria Dascalu, Mohsen Dashti, Maedeh Dastmardi, Berecha Hundessa Demessa, Biniyam Demisse, Diriba Dereje, Awoke Masrie Asrat Derese, Nikolaos Dervenis, Vinoth Gnana Chellaiyan Devanbu, Thanh Chi Do, Thao Huynh Phuong Do, Francisco Winter dos Santos Figueiredo, Arkadiusz Marian Dziedzic, Hisham Atan Edinur, Ferry Efendi, Joshua R. Ehrlich, Michael Ekholuenetale, Temitope Cyrus Ekundayo, Iman El Sayed, Muhammed Elhadi, Mohammad Hassan Emamian, Mehdi Emamverdi, Azin Etemadimanesh, Adeniyi Francis Fagbamigbe, Ayesha Fahim, Hossein Farrokhpour, Ali Fatehizadeh, Alireza Feizkhah, Lorenzo Ferro Desideri, Getahun Fetensa, Florian Fischer, Ali Forouhari, Matteo Foschi, Kayode Raphael Fowobaje, Abhay Motiramji Gaidhane, Aravind P. Gandhi, Miglas W. W. Gebregergis, Mesfin Gebrehiwot, Brhane Gebremariam, Urge Gerema, Fariba Ghassemi, Sherief Ghozy, Mahaveer Golechha, Pouya Goleij, Bárbara Niegia Garcia Goulart, Shi-Yang Guan, Zewdie Gudisa, Sapna Gupta, Veer Bala Gupta, Vivek Kumar Gupta, Arvin Haj-Mirzaian, Aram Halimi, Shahin Hallaj, Samer Hamidi, Mehdi Harorani, Hamidreza Hasani, Demisu Zenbaba Heyi, Nguyen Quoc Hoan, Ramesh Holla, Sung Hwi Hong, Mehdi Hosseinzadeh, Chengxi Hu, John J. Huang, Hong-Han Huynh, Segun Emmanuel Ibitoye, Irena M. Ilic, Mustapha Immurana, Md. Rabiul Islam, Sheikh Mohammed Shariful Islam, Chidozie C. D. Iwu, Louis Jacob, Ammar Abdulrahman Jairoun, Manthan Dilipkumar Janodia, Shubha Jayaram, Har Ashish Jindal, Mohammad Jokar, Nitin Joseph, Charity Ehimwenma Joshua, Vidya Kadashetti, Laleh R. Kalankesh, Rohollah Kalhor, Sagarika Kamath, Himal Kandel, Rami S. Kantar, Ibraheem M. Karaye, Hengameh Kasraei, Soujanya Kaup, Navjot Kaur, Rimple Jeet Kaur, Gbenga A. Kayode, Yousef Saleh Khader, Himanshu Khajuria, Rovshan Khalilov, Mahalaqua Nazli Khatib, Adnan Kisa, Soewarta Kosen, Ai Koyanagi, Kewal Krishan, Mukhtar Kulimbet, Nithin Kumar, Om P. Kurmi, Chandrakant Lahariya, Tuo Lan, Iván Landires, Janet L. Leasher, Munjae Lee, Seung Won Lee, Wei-Chen Lee, Stephen S. Lim, Julie-Anne Little, Preetam Bhalchandra Mahajan, Sandeep B. Maharaj, Alireza Mahmoudi, Razzagh Mahmoudi, Kashish Malhotra, Tauqeer Hussain Mallhi, Vahid Mansouri, Emmanuel Manu, Roy Rillera Marzo, Andrea Maugeri, Colm McAlinden, Wondwosen Mebratu, Tesfahun Mekene Meto, Yang Meng, Abera M. Mersha, Tomislav Mestrovic, Le Huu Nhat Minh, Awoke Misganaw, Manish Mishra, Sanjeev Misra, Nouh Saad Mohamed, Soheil Mohammadi, Mustapha Mohammed, Hoda Mojiri-forushani, Ali H. Mokdad, Hossein Molavi Vardanjani, Mohammad Ali Moni, Fateme Montazeri, Maryam Moradi, Rohith Motappa, Parsa Mousavi, Admir Mulita, Christopher J. L. Murray, Ganesh R. Naik, Gurudatta Naik, Shumaila Nargus, Zuhair S. Natto, Biswa Prakash Nayak, Mohammad Negaresh, Hadush Negash, Dang H. Nguyen, Phat Tuan Nguyen, Van Thanh Nguyen, Robina Khan Niazi, Osaretin Christabel Okonji, Andrew T. Olagunju, Matthew Idowu Olatubi, Michal Ordak, Uchechukwu Levi Osuagwu, Nikita Otstavnov, Mayowa O. Owolabi, Jagadish Rao Padubidri, Ashok Pandey, Georgios D. Panos, Shahina Pardhan, Seoyeon Park, Jay Patel, Shrikant Pawar, Prince Peprah, Ionela-Roxana Petcu, Alireza Peyman, Hoang Tran Pham, Mohsen Pourazizi, Nguyen Khoi Quan, Fakher Rahim, Vafa Rahimi-Movaghar, Mohammad Hifz Ur Rahman, Sathish Rajaa, Shakthi Kumaran Ramasamy, Premkumar Ramasubramani, Shubham Ranjan, Mohammad-Mahdi Rashidi, Rama Shankar Rath, Annisa Utami Rauf, Salman Rawaf, Amirmasoud Rayati Damavandi, Elrashdy Moustafa Mohamed Redwan, Priyanka Roy, Koushik Roy Pramanik, Zahra Saadatian, Siamak Sabour, Basema Saddik, Umar Saeed, Sare Safi, Sher Zaman Safi, Amene Saghazadeh, Fatemeh Saheb Sharif-Askari, Amirhossein Sahebkar, Mohammad Ali Sahraian, Joseph W. Sakshaug, Mohamed A. Saleh, Sara Samadzadeh, Yoseph Leonardo Samodra, Vijaya Paul Samuel, Abdallah M. Samy, Aswini Saravanan, Siddharthan Selvaraj, Farbod Semnani, Sabyasachi Senapati, Yashendra Sethi, Seyed Arsalan Seyedi, Allen Seylani, Amira A. Shaheen, Samiah Shahid, Moyad Jamal Shahwan, Masood Ali Shaikh, Sunder Sham, Muhammad Aaqib Shamim, Mohammed Shannawaz, Bereket Beyene Shashamo, Maryam Shayan, Aminu Shittu, Ivy Shiue, K. M. Shivakumar, Seyed Afshin Shorofi, Migbar Mekonnen Sibhat, Emmanuel Edwar Siddig, Juan Carlos Silva, Jasvinder A. Singh, Paramdeep Singh, Eirini Skiadaresi, Yonatan Solomon, Raúl A. R. C. Sousa, Chandrashekhar T. Sreeramareddy, Vladimir I. Starodubov, Mohana Devi Subramaniam, Sri Susanty, Seyyed Mohammad Tabatabaei, Birhan Tsegaw Taye, Gebrehiwot Teklay, Mohamad-Hani Temsah, Dufera Rikitu Terefa, Jansje Henny Vera Ticoalu, Temesgen Mohammed Toma, Aristidis Tsatsakis, Guesh Mebrahtom Tsegay, Munkhtuya Tumurkhuu, Biruk Shalmeno Tusa, Sree Sudha Ty, Chukwudi S. Ubah, Muhammad Umair, Tungki Pratama Umar, Rohollah Valizadeh, Jef Van den Eynde, Stephanie Louise Watson Watson, Tewodros Eshete Wonde, Guadie Sharew Wondimagegn, Hong Xiao, Yao Yao, Iman Yazdani Nia, Arzu Yiğit, Yazachew Yismaw, Dong Keon Yon, Naohiro Yonemoto, Yuyi You, Chuanhua Yu, Mikhail Sergeevich Zastrozhin, Hanqing Zhao, Makan Ziafati, Magdalena Zielińska, Yossef Teshome Zikarg, Mohammad Zoladl, Jaimie D. Steinmetz

**Affiliations:** 1 Medicine & Health, University of New South Wales, Sydney, NSW Australia; 2 School of Population and Global Health, University of Melbourne, Carlton, VIC Australia; 3 Vision and Eye Research Institute, Anglia Ruskin University, Cambridge, UK; 4 HelpMeSee, Instituto Mexicano de Oftalmologia, Santiago de Querétaro, Mexico; 5 University of Miami, Gables, FL USA; 6 University of Utah, Salt Lake City, UT USA; 7 Department of Ophthalmology, Massachusetts Eye and Ear/Shepens Eye Research Institute/Harvard Medical School, Boston, MA USA; 8 Eye Unit, MyungSung Christian Medical Center (MCM) Comprehensive Specialized Hospital and MyungSun Medical College, Addis Ababa, Ethiopia; 9 Department of Ophthalmology, Addis Ababa University, Addis Ababa, Ethiopia; 10 Sight for Souls, Bellevue, WA USA; 11 Addenbrooke’s Hospital, Cambridge, UK; 12 Federal University of Sao Paolo, Sao Paolo, SP Brazil; 13 University of Calgary, Calgary, AB Canada; 14 School of Medicine, Vita-Salute San Raffaele University, Milan, Italy; 15 Department of Ophthalmology, IRCCS San Raffaele Scientific Institute, Milan, Italy; 16 IRCCS San Raffaele Scientific Institute, Vita-Salute University, Milan, Italy; 17 University of Poitiers, Poitiers, France; 18 CHU de Poitiers, Poitiers, France; 19 Brien Holden Vision Institute, Sydney, NSW Australia; 20 School of Optometry and Vision Sciences, Faculty of Medicine, University of New South Wales, Sydney, NSW Australia; 21 Department of Computer Science, University of Oxford, Oxford, UK; 22 Ufa Eye Research Institute, Ufa, Russia; 23 School of Life Course and Population Sciences, King’s College London, London, UK; 24 The Medical Eye Unit, Guy’s and St Thomas’ NHS Foundation Trust, London, UK; 25 University Hospital, Dijon, France; 26 National University of Singapore, Singapore, Singapore; 27 Singapore Eye Research Institute, Singapore, Singapore; 28 University of Michigan, Ann Arbor, MI USA; 29 Kellogg Eye Center, Ann Arbor, MI USA; 30 Institute for Social Research, University of Michigan, Ann Arbor, MI USA; 31 Department of Ophthalmology and Visual Sciences, University of Michigan, Ann Arbor, MI USA; 32 National Eye Institute, Bethesda, MD USA; 33 Mass Eye and Ear, Harvard Medical School, Boston, MA USA; 34 Ribeirão Preto Medical School, University of São Paulo, Sao Paulo, Brazil; 35 Institute of Ophthalmology UCL & NIHR Biomedical Research Centre, London, UK; 36 Sankara Nethralaya, Medical Research Foundation, Chennai, 600006 India; 37 Stanford University, Stanford, CA USA; 38 Department of Ophthalmology, Medical Faculty Mannheim, Heidelberg University, Heidelberg, Germany; 39 Associated Ophthalmologists of Monastir, Monastir, Tunisia; 40 Fattouma Bourguiba University Hospital, University of Monastir, Monastir, Tunisia; 41 Allen Foster Community Eye Health Research Centre, Gullapalli Pratibha Rao International Centre for Advancement of Rural Eye care, L.V. Prasad Eye Institute, Hyderabad, India; 42 Brien Holden Eye Research Centre, L.V. Prasad Eye Institute, Banjara Hills, Hyderabad, India; 43 School of Optometry and Vision Science, University of New South Wales, Sydney, NSW Australia; 44 University of Rochester, School of Medicine and Dentistry, Rochester, NY USA; 45 Nova Southeastern University College for Optometry, Fort Lauderdale, FL USA; 46 Ulster University, Coleraine, UK; 47 Suraj Eye Institute, Nagpur, India; 48 Institute of Optics and Optometry, University of Social Science, 121 Gdanska street, Lodz, 90-519 Poland; 49 Centre for Public Health, Queens University Belfast, Belfast, Northern Ireland UK; 50 John Hopkins Wilmer Eye Institute, Baltimore, USA; 51 1st Department of Ophthamology, Medical School, Aristotle University of Thessaloniki, Ahepa Hospital, Thessaloniki, Greece; 52 University of Crete Medical School, Giofirakia, Greece; 53 Beijing Institute of Ophthamology, Beijing Tongren Hospital, Capital Medical University, Beijing Ophthamology and Visual Sciences Key Laboratory, Beijing, China; 54 Beijing Institute of Ophthamology, Beijing Tongren Eye Center, Beijing Tongren Hospital, Capital Medical University, Beijing, China; 55 Chief Medical Office, HelpMeSee, New York, NY USA; 56 Mexican Institute of Ophthalmology, Queretaro, Mexico; 57 Department of Ophthalmology, Harvard University, Boston, MA USA; 58 Eye Unit, MyungSung Medical College, Addis Ababa, Ethiopia; 59 Department of Ophthalmology, Cambridge University Hospitals, Cambridge, UK; 60 Department of Ophthalmology and Visual Sciences, Federal University of São Paulo, Sao Paulo, Brazil; 61 Department of Ophthalmology, San Raffaele Scientific Institute, Milano, Italy; 62 Scientific Institute San Raffaele Hospital, Vita-Salute University, Milan, Italy; 63 Ophthalmology Department, CHU de Poitiers (Poitiers University Hospital), Poitiers, France; 64 Unité 1084, National Institute of Health and Medical Research (INSERM), Poitiers, France; 65 Institute for Health Metrics and Evaluation, University of Washington, Seattle, WA USA; 66 Department of Health Metrics Sciences, School of Medicine, University of Washington, Seattle, WA USA; 67 Department of Mathematics, Imperial College London, London, UK; 68 Department of Clinical Governance and Quality Improvement, Aleta Wondo Hospital, Aleta Wondo, Ethiopia; 69 The Institute of Pharmaceutical Sciences (TIPS), Tehran University of Medical Sciences, Tehran, Iran; 70 School of Pharmacy, Tehran University of Medical Sciences, Tehran, Iran; 71 School of Medicine, Shiraz University of Medical Sciences, Shiraz, Iran; 72 Pediatrics Nursing Department, Debre Berhan University, Debre Berhan, Ethiopia; 73 Department of Community Medicine, Babcock University, Ilishan-Remo, Nigeria; 74 Department of Family and Community Health, University of Health and Allied Sciences, Ho, Ghana; 75 Department of Adult Health Nursing, Aksum University, Aksum, Ethiopia; 76 Department of Nursing, Al Zaytoonah University of Jordan, Amman, Jordan; 77 Department of Banking and Finance, University of Human Development, Sulaymaniyah, Iraq; 78 Clinical Sciences Department, University of Sharjah, Sharjah, United Arab Emirates; 79 Department of Therapeutics, United Arab Emirates University, Al Ain, United Arab Emirates; 80 College of Pharmacy, University of Jordan, Amman, Jordan; 81 Department of Public Health, Wolkite University, Wolkite, Ethiopia; 82 College of Medicine and Health Sciences, Bahir Dar University, Bahir Dar, Ethiopia; 83 Centre for Social Research in Health, University of New South Wales, Sydney, NSW Australia; 84 Quality and Systems Performance Unit, Cancer Institute NSW, Sydney, NSW Australia; 85 Faculty of Medicine, Universitas Padjadjaran (Padjadjaran University), Bandung, Indonesia; 86 Department of Life Sciences, University of Management and Technology, Lahore, Pakistan; 87 Department of Biotechnology, Shahid Beheshti University of Medical Sciences, Tehran, Iran; 88 School of Public Health, University of Technology Sydney, Sydney, NSW Australia; 89 Department of Medical Biochemistry, Shaqra University, Shaqra, Saudi Arabia; 90 Department of Health and Biological Sciences, Abasyn University, Peshawar, Pakistan; 91 Department of Natural Sciences, Labanese American University, Beirut, Lebanon; 92 Department of Epidemiology and Biostatistics, Shahrekord University of Medical Sciences, Shahrekord, Iran; 93 Department of Epidemiology, Shahid Beheshti University of Medical Sciences, Tehran, Iran; 94 Institute of Endemic Diseases, University of Khartoum, Khartoum, Sudan; 95 Swiss Tropical and Public Health Institute, University of Basel, Basel, Switzerland; 96 Department of Biosciences, COMSATS Institute of Information Technology, Islamabad, Pakistan; 97 Department of Ophthalmology, University of Leipzig Medical Center, Leipzig, Germany; 98 Department of Ophthalmology, Charité Universitätsmedizin Berlin (Charité Medical University Berlin), Berlin, Germany; 99 Department of Zoology, Abdul Wali Khan University Mardan, Mardan, Pakistan; 100 Center for Biotechnology and Microbiology, University of SWAT, Swat, Pakistan; 101 Centre for Research in Molecular Medicine, Institute of Molecular Biology and Biotechnology, Lahore, Pakistan; 102 Department of Population and Behavioural Sciences, University of Health and Allied Sciences, Ho, Ghana; 103 Department of Medicine, University of Thessaly, Volos, Greece; 104 Department of Ophthalmology, Inselspital, Bern, Switzerland; 105 Department of Vitreoretinal, Moorfields Eye Hospital, London, UK; 106 Department of Pharmacology, All India Institute of Medical Sciences, Jodhpur, India; 107 All India Institute of Medical Sciences, Bhubaneswar, India; 108 Regenerative Medicine, Organ Procurement and Transplantation Multi-diciplinary Center, Guilan University of Medical Sciences, Rasht, Iran; 109 School of Dentistry and Medical Sciences, Charles Sturt University, Orange, NSW Australia; 110 Department of Social Sciences, Berekum College of Education, Berekum, Ghana; 111 School of Public Health, Kwame Nkrumah University of Science and Technology, Kumasi, Ghana; 112 Health Management and Economics Research Center, Iran University of Medical Sciences, Tehran, Iran; 113 College of Pharmacy, Al Ain University, Abu Dhabi, United Arab Emirates; 114 College of Art and Science, Ottawa University, Surprise, AZ USA; 115 School of Life Sciences, Arizona State University, Tempe, AZ USA; 116 Department of Anatomy, Shiraz University of Medical Sciences, Shiraz, Iran; 117 College of Medicine and Health Sciences, Adigrat University, Adigrat, Ethiopia; 118 Department of Environmental Health, Mekelle University, Mekelle, Ethiopia; 119 University Institute of Radiological Sciences and Medical Imaging Technology, The University of Lahore, Lahore, Pakistan; 120 Department of Immunology, Zanjan University of Medical Sciences, Zanjan, Iran; 121 School of Nursing and Midwifery, Debre Berhan University, Debre Berhan, Ethiopia; 122 Faculty of Nursing, Philadelphia University, Amman, Jordan; 123 Department of Forensic Medicine, Lumbini Medical College, Palpa, Nepal; 124 Department of Health Information Management, Iran University of Medical Sciences, Tehran, Iran; 125 Department of Neurovascular Research, Nested Knowledge, Inc, Saint Paul, MN USA; 126 Faculty of Medicine, October 6 University, 6th of October City, Egypt; 127 Department of Nursing, Semnan University of Medical Sciences and Health Services, Semnan, Iran; 128 School of Medicine, Isfahan University of Medical Sciences, Isfahan, Iran; 129 Department of Community Medicine and Family Medicine, All India Institute of Medical Sciences, Rishikesh, India; 130 University Institute of Public Health, The University of Lahore, Lahore, Pakistan; 131 Institute of Health and Wellbeing (IHW), Federation University Australia, Melbourne, VC Australia; 132 Manna Institute, University of New England, Armidale, NSW Australia; 133 Miami Cancer Institute, Baptist Health South Florida, Miami, FL USA; 134 Department of Academics, Indian Institute of Public Health, Gurgaon, India; 135 Department of Medical Education, University of Nevada, Las Vegas, Las Vegas, NV USA; 136 Department of Surgery, Jimma University, jimma, Ethiopia; 137 Farabi Eye Hospital, Tehran University of Medical Sciences, Tehran, Iran; 138 School of Medicine and Public Health, University of Newcastle, Newcastle, NSW Australia; 139 School of Public Health, Haramaya University, Harar, Ethiopia; 140 Department of Forensic Chemistry, Government Institute of Forensic Science, Aurangabad, Aurangabad, India; 141 Department of Public Health, North Dakota State University, Fargo, ND USA; 142 Department of Community Medicine and Family Medicine, All India Institute of Medical Sciences, Jodhpur, India; 143 School of Public Health, All India Institute of Medical Sciences, Jodhpur, India; 144 Global Health Neurology Lab, NSW Brain Clot Bank, Sydney, NSW Australia; 145 Department of Neurology and Neurophysiology, South West Sydney Local Heath District and Liverpool Hospital, Sydney, NSW Australia; 146 Human Genetics and Molecular Medicine, Central University of Punjab, Bathinda, India; 147 Epidemiology Department, Ufa Eye Research Institute, Ufa, Russia; 148 Department of Ophthalmology, Shahid Beheshti University of Medical Sciences, Tehran, Iran; 149 Department of Medicine, Division of Clinical Epidemiology, McGill University, Montreal, QC Canada; 150 Centre for Outcomes Research and Evaluation, Research Institute of the McGill University Health Centre, Montreal, QC Canada; 151 Faculty of Health Sciences, University of Botswana, Gaborone, Botswana; 152 Ophthalmology Department, Moorfields Eye Hospital NHS Foundation Trust, London, UK; 153 International Centre for Eye Health, London School of Hygiene & Tropical Medicine, London, UK; 154 Department of Biopharmaceutics and Clinical Pharmacy, The University of Jordan, Amman, Jordan; 155 Department of Basic Biomedical Sciences, University of Sharjah, Sharjah, United Arab Emirates; 156 School of Public Health and Health Systems, University of Waterloo, Waterloo, ON Canada; 157 Al Shifa School of Public Health, Al Shifa Trust Eye Hospital, Rawalpindi, Pakistan; 158 Harvard Business School, Harvard University, Boston, MA USA; 159 Internal Medicine Department, Hospital Italiano de Buenos Aires (Italian Hospital of Buenos Aires), Buenos Aires, Argentina; 160 Board of Directors, Argentine Society of Medicine, Buenos Aires, Argentina; 161 School of Sciences, University of Minho, Braga, Portugal; 162 Association of Licensed Optometry Professionals, Linda-a-Velha, Portugal; 163 College of Public Health, Medical and Veterinary Sciences, James Cook University, Townsville, QLD Australia; 164 Public Health Departement, University of Mataram, Mataram, Indonesia; 165 Department of Anesthesiology and Perioperative Medicine, University of Rochester, Rochester, NY USA; 166 Temerty Faculty of Medicine, University of Toronto, Toronto, ON Canada; 167 Saveetha Dental College, Saveetha University, Chennai, India; 168 Macquarie Medical School, Macquarie University, Sydney, NSW Australia; 169 Chitkara College of Pharmacy, Chitkara Univeristy, Punjab, India; 170 Center for Biomedicine and Community Health, VNU-International School, Hanoi, Viet Nam; 171 University Hospital Center of Porto, University of Porto, Porto, Portugal; 172 Therapeutic and Diagnostic Technologies, Cooperativa de Ensino Superior Politécnico e Universitário (Polytechnic and University Higher Education Cooperative), Gandra, Portugal; 173 Institute for Research and Innovation in Health, University of Porto, Porto, Portugal; 174 Department of Addiction Medicine, Haukland University Hospital, Bergen, Norway; 175 Department of Global Public Health and Primary Care, University of Bergen, Bergen, Norway; 176 Ingram School of Engineering, Texas State University, San Marcos, TX USA; 177 Ophthalmology Department, Carol Davila University of Medicine and Pharmacy, Bucharest, Romania; 178 Ophthalmology Department, Emergency University Hospital Bucharest, Bucuresti, Romania; 179 Department of Radiology, Tabriz University of Medical Sciences, Tabriz, Iran; 180 Iran University of Medical Sciences, Tehran, Iran; 181 Tehran University of Medical Science, Tehran University of Medical Sciences, Tehran, Iran; 182 USAID-JSI, Jimma University, Addis Ababa, Ethiopia; 183 Department of Nursing, Arba Minch University, Arba Minch, Ethiopia; 184 Department of Biomedical Sciences, Jimma University, Jimma, Ethiopia; 185 St Paul’s Eye Unit, Royal Liverpool University Hospital, Liverpool, UK; 186 Department of Ophthalmology, Aristotle University of Thessaloniki, Thessaloniki, Greece; 187 Department of Community Medicine, Chettinad Hospital and Research Institute, Chennai, India; 188 Department of Medicine, Pham Ngoc Thach University of Medicine, Ho Chi Minh City, Viet Nam; 189 Department of Medicine, Can Tho University of Medicine and Pharmacy, Can Tho, Viet Nam; 190 Epidemiology and Data Analysis Laboratory, University Center FMABC, Santo André, Brazil; 191 Department of Conservative Dentistry with Endodontics, Medical University of Silesia, Katowice, Poland; 192 School of Health Sciences, Universiti Sains Malaysia (University of Science Malaysia), Kubang Kerian, Malaysia; 193 Advanced Nursing Department, Universitas Airlangga, Surabaya, Indonesia; 194 Department of Ophthalmology and Visual Sciences, University of Michigan, Ann Arbor, GA USA; 195 Institute for Health Care Policy and Innovation, University of Michigan, Ann Arbor, MI USA; 196 Department of Epidemiology and Medical Statistics, University of Ibadan, Ibadan, Nigeria; 197 Faculty of Public Health, University of Ibadan, Ibadan, Nigeria; 198 Department of Biological Sciences, University of Medical Sciences, Ondo, Ondo Nigeria; 199 Biomedical Informatics and Medical Statistics Department, Alexandria University, Alexandria, Egypt; 200 Faculty of Medicine, University of Tripoli, Tripoli, Libya; 201 Ophthalmic Epidemiology Research Center, Shahroud University of Medical Sciences, Shahroud, Iran; 202 Department of Ophthalmology, University of California Los Angeles, Los Angeles, CA USA; 203 Department of Physical Medicine and Rehabilitation, Johns Hopkins University, Baltimore, MD USA; 204 Research Centre for Healthcare and Community, Coventry University, Coventry, UK; 205 Department of Oral Biology, The University of Lahore, Lahore, Pakistan; 206 School of Medicine, Tehran University of Medical Sciences, Tehran, Iran; 207 Endocrinology and Metabolism Research Institute, Non-Communicable Diseases Research Center (NCDRC), Tehran, Iran; 208 Department of Environmental Health Engineering, Isfahan University of Medical Sciences, Isfahan, Iran; 209 Department of Social Medicine and Epidemiology, Guilan University of Medical Sciences, Rasht, Iran; 210 University Eye Clinic, University of Genoa, Genoa, Italy; 211 Department of Nursing, Wollega University, Nekemte, Ethiopia; 212 Institute of Public Health, Charité Universitätsmedizin Berlin (Charité Medical University Berlin), Berlin, Germany; 213 Department of Ophthalmology, Isfahan University of Medical Sciences, Isfahan, Iran; 214 Emergency Department, Isfahan University of Medical Sciences, Isfahan, Iran; 215 Department of Biotechnological and Applied Clinical Sciences (DISCAB), Multiple Sclerosis Research Center, L’Aquila, Italy; 216 Department of Neuroscience, Multiple Sclerosis Research Center, Ravenna, Italy; 217 Child Survival Unit, Centre for African Newborn Health and Nutrition, Ibadan, Nigeria; 218 Department of Community Medicine, Datta Meghe Institute of Medical Sciences, Wardha, India; 219 Department of Community Medicine, ESIC Medical College & Hospital, Hyderabad, India; 220 Department of Midwifery, Adigrat University, Adigrat, Ethiopia; 221 Department of Environmental Health, Wollo University, Dessie, Ethiopia; 222 Department of Health, Policy Research Institute, Mekelle, Ethiopia; 223 Simon Fraser University, Mekelle, Ethiopia; 224 Department of Public Health, Jimma University, Jimma, Ethiopia; 225 Department of Ophthalmology, Tehran University of Medical Sciences, Tehran, Iran; 226 Department of Radiology, Mayo Clinic, Rochester, MN USA; 227 Health Systems and Policy Research Department, Indian Institute of Public Health, Gandhinagar, India; 228 Department of Genetics, Sana Institute of Higher Education, Sari, Iran; 229 Universal Scientific Education and Research Network (USERN), Kermanshah University of Medical Sciences, Kermanshah, Iran; 230 Postgraduate Program in Epidemiology, Federal University of Rio Grande do Sul, Porto Alegre, Brazil; 231 Department of Epidemiology and Biostatistics, Anhui Medical University, Hefei, China; 232 Department of Anesthesia, Madda Walabu University, Goba, Ethiopia; 233 Toxicology Department, Shriram Institute for Industrial Research, Delhi, India; 234 School of Medicine, Deakin University, Geelong, VIC Australia; 235 Faculty of Medicine Health and Human Sciences, Macquarie University, Sydney, NSW Australia; 236 Department of Radiology, Massachusetts General Hospital, Boston, MA USA; 237 Obesity Research Center, Shahid Beheshti University of Medical Sciences, Tehran, Iran; 238 Research Institute for Endocrine Sciences, Shahid Beheshti University of Medical Sciences, Tehran, Iran; 239 Department of Ophthalmology, Thomas Jefferson University, Philadelphia, PA USA; 240 Department of Ophthalmology, University of California San Diego, La Jolla, CA USA; 241 School of Health and Environmental Studies, Hamdan Bin Mohammed Smart University, Dubai, United Arab Emirates; 242 Department of Nursing, Arak University of Medical Sciences, Arak, Iran; 243 Department of Ophthalmology, Iran University of Medical Sciences, Karaj, Iran; 244 Department of Public Health, Madda Walabu University, Goba, Ethiopia; 245 School of Dentistry, Hanoi Medical University, Hanoi, Viet Nam; 246 Kasturba Medical College, Mangalore, Manipal Academy of Higher Education, Manipal, India; 247 Department of Pediatrics, Yonsei University College of Medicine, Seoul, South Korea; 248 Research Department, Electronic Medical Records for the Developing World, York, UK; 249 Institute of Research and Development, Duy Tan University, Da Nang, Viet Nam; 250 Department of Computer Science, University of Human Development, Sulaymaniyah, Iraq; 251 Department of Psychology, Tsinghua University, Beijing, China; 252 Department of Ophthalmology and Visual Science, Yale University, New Haven, CT USA; 253 School of Biotechnology, Tan Tao University, Long An, Viet Nam; 254 Department of Health Promotion and Education, University of Ibadan, Ibadan, Nigeria; 255 Faculty of Medicine, University of Belgrade, Belgrade, Serbia; 256 Institute of Health Research, University of Health and Allied Sciences, Ho, Ghana; 257 Department of Pharmacy, University of Asia Pacific, Dhaka, Bangladesh; 258 Institute for Physical Activity and Nutrition, Deakin University, Burwood, VIC Australia; 259 Sydney Medical School, University of Sydney, Sydney, NSW Australia; 260 School of Health Systems and Public Health, University of Pretoria, Pretoria, South Africa; 261 Research and Development Unit, Biomedical Research Networking Center for Mental Health Network (CiberSAM), Sant Boi de Llobregat, Spain; 262 Faculty of Medicine, University of Versailles Saint-Quentin-en-Yvelines, Montigny-le-Bretonneux, France; 263 Department of Clinical Pharmacy, University of Science Malaysia, Penang, Malaysia; 264 Department of Health and Safety, United Arab Emirates University, Dubai, United Arab Emirates; 265 Manipal College of Pharmaceutical Sciences, Manipal Academy of Higher Education, Manipal, India; 266 Department of Biochemistry, Government Medical College, Mysuru, India; 267 National Health System Resource Centre, Ministry of Health & Family Welfare, New Delhi, India; 268 Zoonoses Research Center, Islamic Azad University, Karaj, Iran; 269 Department of Clinical Sciences, Jahrom University of Medical Sciences, Jahrom, Iran; 270 Department of Community Medicine, Manipal Academy of Higher Education, Mangalore, India; 271 Department of Economics, National Open University, Benin City, Nigeria; 272 Department of Oral and Maxillofacial Pathology, Krishna Vishwa Vidyapeeth (Deemed to be University), Karad, India; 273 Social Determinants of Health Research Center, Gonabad University of Medical Sciences, Gonabad, Iran; 274 Institute for Prevention of Non-communicable Diseases, Qazvin University of Medical Sciences, Qazvin, Iran; 275 Health Services Management Department, Qazvin University of Medical Sciences, Qazvin, Iran; 276 Manipal Institute of Management, Manipal Academy of Higher Education, Manipal, India; 277 Save Sight Institute, University of Sydney, Sydney, NSW Australia; 278 Sydney Eye Hospital, South Eastern Sydney Local Health District, Sydney, NSW Australia; 279 The Hansjörg Wyss Department of Plastic and Reconstructive Surgery, Nab’a Al-Hayat Foundation for Medical Sciences and Health Care, New York, NY USA; 280 Department of Cleft Lip and Palate Surgery, Global Smile Foundation, Norwood, MA USA; 281 School of Health Professions and Human Services, Hofstra University, Hempstead, NY USA; 282 Department of Anesthesiology, Montefiore Medical Center, Bronx, NY USA; 283 Eye Research Center, Iran University of Medical Sciences, Tehran, Iran; 284 Health Policy Research Center, Shiraz University of Medical Sciences, Shiraz, Iran; 285 Department of Ophthalmology, Yenepoya Medical College, Mangalore, India; 286 Department of ENT, Dr. B. R. Ambedkar State Institute of Medical Sciences (AIMS), Mohali, India; 287 International Research Center of Excellence, Institute of Human Virology Nigeria, Abuja, Nigeria; 288 Julius Centre for Health Sciences and Primary Care, Utrecht University, Utrecht, The Netherlands; 289 Department of Public Health, Jordan University of Science and Technology, Irbid, Jordan; 290 Amity Institute of Forensic Sciences, Amity University, Noida, India; 291 Department of Biophysics and Biochemistry, Baku State University, Baku, Azerbaijan; 292 Azerbaijan State University of Economics (UNEC), Baku, Azerbaijan; 293 Global Consortium for Public Health Research, Datta Meghe Institute of Higher Education and Research, Wardha, India; 294 School of Health Sciences, Kristiania University College, Oslo, Norway; 295 Department of International Health and Sustainable Development, Tulane University, New Orleans, LA USA; 296 Independent Consultant, Jakarta, Indonesia; 297 San Juan de Dios Sanitary Park, Barcelona, Spain; 298 Department of Anthropology, Panjab University, Chandigarh, India; 299 Department of Health Research, Almaty, Kazakhstan; 300 Atchabarov Scientific Research Institute of Fundamental and Applied Medicine, Kazakh National Medical University, Almaty, Kazakhstan; 301 Faculty of Health and Life Sciences, Coventry University, Coventry, UK; 302 Department of Medicine, McMaster University, Hamilton, ON Canada; 303 Department of Health Policy and Strategy, Foundation for People-centric Health Systems, New Delhi, India; 304 SD Gupta School of Public Health, Indian Institute of Health Management Research University, Jaipur, India; 305 Department of Surgery, Washington University in St. Louis, Saint Louis, MO USA; 306 Unit of Genetics and Public Health, Institute of Medical Sciences, Las Tablas, Panama; 307 Ministry of Health, Herrera, Panama; 308 College of Optometry, Nova Southeastern University, Fort Lauderdale, FL USA; 309 Department of Medical Humanities and Social Medicine, Ajou University School of Medicine, Suwon, South Korea; 310 Medial Research Collaborating Center, Ajou University Medical Center, Suwon, South Korea; 311 Department of Precision Medicine, Sungkyunkwan University, Suwon-si, South Korea; 312 Department of Internal Medicine, University of Texas, Galveston, TX USA; 313 School of Biomedical Sciences, Coleraine, UK; 314 Department of Community Medicine, Jawaharlal Institute of Postgraduate Medical Education and Research, Karaikal, India; 315 School of Pharmacy, University of the West Indies, St. Augustine, Trinidad and Tobago; 316 Fellow, Planetary Health Alliance, Boston, MA USA; 317 Department of Food Hygiene and Safety, Qazvin University of Medical Sciences, Qazvin, Iran; 318 Department of Internal Medicine, Dayanand Medical College and Hospital, Ludhiana, India; 319 Department of Clinical Pharmacy, Jouf University, Sakaka, Saudi Arabia; 320 Digestive Diseases Research Institute, Tehran University of Medical Sciences, Tehran, Iran; 321 Department of Public Health, Management and Science University, Shah Alam, Malaysia; 322 Jeffrey Cheah School of Medicine and Health Sciences, Monash University, Subang Jaya, Malaysia; 323 Department GF Ingrassia, University of Catania, Catania, Italy; 324 Department of Ophthalmology, Princess of Wales Hospital, Wales, UK; 325 School of Optometry and Vision Sciences, Cardiff University, Cardiff, UK; 326 Department of Epidemiology and Biostatistics, Wollo University, Dessie, Ethiopia; 327 Department of Research, Performance Monitoring and Accountability 2020-Ethiopia, Addis Ababa, Ethiopia; 328 Department of Public Health, Arba Minch University, Arba Minch, Ethiopia; 329 Eye Center, Wuhan University, Wuhan, China; 330 University Centre Varazdin, University North, Varazdin, Croatia; 331 International Ph.D. Program in Medicine, Taipei Medical University, Taipei, Taiwan, ROC; 332 Research Center for Artificial Intelligence in Medicine, Taipei Medical University, Taipei, Taiwan, ROC; 333 National Data Management Center for Health, Ethiopian Public Health Institute, Addis Ababa, Ethiopia; 334 Department of Biomedical Sciences, Mercer University School of Medicine, Macon, GA USA; 335 Department of Surgical Oncology, All India Institute of Medical Sciences, Jodhpur, India; 336 Molecular Biology Unit, Sirius Training and Research Centre, Khartoum, Sudan; 337 Bio-Statistical and Molecular Biology Department, Sirius Training and Research Centre, Khartoum, Sudan; 338 Department of Clinical Pharmacy and Pharmacy Practice, Ahmadu Bello University, Zaria, Nigeria; 339 School of Pharmaceutical Sciences, Universiti Sains Malaysia (University of Science Malaysia), Penang, Malaysia; 340 Department of Pharmacology, Abadan School of Medical Sciences, Abadan, Iran; 341 Department of Biostatistics, Shiraz University of Medical Sciences, Shiraz, Iran; 342 School of Health & Rehabilitation Sciences, The University of Queensland, Brisbane, QLD Australia; 343 Non-communicable Diseases Research Center, Tehran University of Medical Sciences, Tehran, Iran; 344 School of Medicine, Shahid Beheshti University of Medical Sciences, Tehran, Iran; 345 Department of Medicine, Democritus University of Thrace, Alexandroupolis, Greece; 346 College of Medicine and Public Health, Flinders University, Adelaide, FA Australia; 347 Department of Engineering, Western Sydney University, Sydney, NSW Australia; 348 Comprehensive Cancer Center, University of Alabama at Birmingham, Birmingham, AL USA; 349 Department of Dental Public Health, King Abdulaziz University, Jeddah, Saudi Arabia; 350 Department of Health Policy and Oral Epidemiology, Harvard University, Boston, MA USA; 351 Independent Consultant, Tehran, Iran; 352 Department of Internal Medicine, Ardabil University of Medical Science, Ardabil, Iran; 353 Department of Medical Laboratory Sciences, Adigrat University, Adigrat, Ethiopia; 354 Division of Cardiology, Massachusetts General Hospital, Boston, MA USA; 355 Department of Medical Engineering, University of South Florida, Tampa, FL USA; 356 Department of Surgery, Danang Family Hospital, Danang, Viet Nam; 357 Department of General Medicine, University of Medicine and Pharmacy at Ho Chi Minh City, Ho Chi Minh City, Viet Nam; 358 International Islamic University Islamabad, Islamabad, Pakistan; 359 School of Pharmacy, University of the Western Cape, Cape Town, South Africa; 360 Department of Psychiatry and Behavioural Neurosciences, McMaster University, Hamilton, ON Canada; 361 Department of Psychiatry, University of Lagos, Lagos, Nigeria; 362 Department of Nursing Science, Bowen University, Iwo, Nigeria; 363 Department of Pharmacotherapy and Pharmaceutical Care, Medical University of Warsaw, Warsaw, Poland; 364 School of Medicine, Western Sydney University, Campbelltown, NSW Australia; 365 Department of Optometry and Vision Science, University of KwaZulu-Natal, KwaZulu-Natal, South Africa; 366 Laboratory of Public Health Indicators Analysis and Health Digitalization, Moscow Institute of Physics and Technology, Dolgoprudny, Russia; 367 Department of Medicine, University of Ibadan, Ibadan, Nigeria; 368 Department of Medicine, University College Hospital, Ibadan, Ibadan, Nigeria; 369 Department of Forensic Medicine and Toxicology, Kasturba Medical College, Mangalore, Mangalore, India; 370 Research Department, Nepal Health Research Council, Kathmandu, Nepal; 371 Research Department, Public Health Research Society Nepal, Kathmandu, Nepal; 372 Department of Ophthalmology, Nottingham University Hospitals, QMC Campus, Nottingham, UK; 373 Division of Ophthalmology & Visual Sciences, University of Nottingham, Nottingham, UK; 374 Yonsei University College of Medicine, Seodaemun-gu, South Korea; 375 Global Health Governance Programme, University of Edinburgh, Edinburgh, UK; 376 School of Dentistry, University of Leeds, Leeds, UK; 377 Department of Genetics, Yale University, New Haven, CT USA; 378 Centre for Primary Health Care and Equity, University of New South Wales, Kensington, NSW Australia; 379 Department of Statistics and Econometrics, Bucharest University of Economic Studies, Bucharest, Romania; 380 School of Medicine, Pham Ngoc Thach University of Medicine, Ho Chi Minh City, Viet Nam; 381 College of Health Sciences, VinUniversity, Hanoi, Viet Nam; 382 Department of Health Sciences, Cihan University Sulaimaniya, Sulaymaniyah, Iraq; 383 Cihan University Sulaimaniya Research Center (CUSRC), Sulaymaniyah, Iraq; 384 Sina Trauma and Surgery Research Center, Tehran University of Medical Sciences, Tehran, Iran; 385 Manipal TATA Medical College, Manipal Academy of Higher Education, Manipal, India; 386 Department of Community Medicine, Employees’ State Insurance Model Hospital, Chennai, India; 387 Department of Radiology, Stanford University, Stanford, CA USA; 388 Department of Community Medicine, Mahatma Gandhi Medical College and Research Institute, Puducherry, India; 389 School of Humanities and Social Sciences, Indian Institute of Technology Mandi, Mandi, India; 390 Social Determinants of Health Research Center, Shahid Beheshti University of Medical Sciences, Tehran, Iran; 391 Department of Community Medicine and Family Medicine, All India Institute of Medical Sciences, Gorakhpur, Gorakhpur, India; 392 Department of Health Behaviour, Environment, and Social Medicine, Gadjah Mada University, Yogyakarta, Indonesia; 393 Department of Primary Care and Public Health, Imperial College London, London, UK; 394 Academic Public Health England, Public Health England, London, UK; 395 Department of Medicine, Tehran University of Medical Sciences, Tehran, Iran; 396 Department Biological Sciences, King Abdulaziz University, Jeddah, Egypt; 397 Department of Protein Research, Research and Academic Institution, Alexandria, Egypt; 398 Department of Labour, Directorate of Factories, Government of West Bengal, Kolkata, India; 399 Department of Biostatistics and Epidemiology, International Institute for Population Sciences, Mumbai, India; 400 Faculty of Medicine, Gonabad University of Medical Sciences, Gonabad, Iran; 401 Infectious Diseases Research Center, Gonabad University of Medical Sciences, gonabad, Iran; 402 Sharjah Institute for Medical Research, University of Sharjah, Sharjah, United Arab Emirates; 403 Multidisciplinary Laboratory Foundation University School of Health Sciences (FUSH), Foundation University, Islamabad, Pakistan; 404 International Center of Medical Sciences Research (ICMSR), Islamabad, Pakistan; 405 Ophthalmic Epidemiology Research Center, Shahid Beheshti University of Medical Sciences, Tehran, Iran; 406 Ophthalmic Research Center, Shahid Beheshti University of Medical Sciences, Tehran, Iran; 407 Faculty of Medicine, Bioscience and Nursing, MAHSA University, Selangor, Malaysia; 408 Interdisciplinary Research Centre in Biomedical Materials (IRCBM), COMSATS Institute of Information Technology, Lahore, Pakistan; 409 Research Center for Immunodeficiencies, Tehran University of Medical Sciences, Tehran, Iran; 410 Sharjah Institute of Medical Sciences, University of Sharjah, Sharjah, United Arab Emirates; 411 Applied Biomedical Research Center, Mashhad University of Medical Sciences, Mashhad, Iran; 412 Biotechnology Research Center, Mashhad University of Medical Sciences, Mashhad, Iran; 413 Multiple Sclerosis Research Center, Tehran University of Medical Sciences, Tehran, Iran; 414 Ludwig Maximilian University of Munich, Munich, Germany; 415 Institute for Employment Research, Nuremberg, Germany; 416 College of Medicine, University of Sharjah, Sharjah, United Arab Emirates; 417 Faculty of Pharmacy, Mansoura University, Mansoura, Egypt; 418 Department of Neurology, Charité Universitätsmedizin Berlin (Charité Medical University Berlin), Berlin, Germany; 419 Department of Neurology, University of Southern Denmark, Odense, Denmark; 420 School of Public Health, Taipei Medical University, Taipei, Taiwan, ROC; 421 Department of Anatomy, Ras Al Khaimah Medical and Health Sciences University, Ras Al Khaimah, United Arab Emirates; 422 Department of Entomology, Ain Shams University, Cairo, Egypt; 423 Medical Ain Shams Research Institute (MASRI), Ain Shams University, Cairo, Egypt; 424 Department of Pharmacology and Research, All India Institute of Medical Sciences, Jodhpur, India; 425 Indira Gandhi Medical College and Research Institute, Puducherry, India; 426 Faculty of Dentistry, AIMST University, Bedong, Malaysia; 427 Faculty of Medicine, Tehran University of Medical Sciences, Tehran, Iran; 428 Department of Human Genetics and Molecular Medicine, Central University of Punjab, Bathinda, India; 429 Department of Medicine and Surgery, Government Doon Medical College, Dehradun, India; 430 Endocrinology and Metabolism Research Center (EMRC), Tehran University of Medical Sciences, Tehran, Iran; 431 National Heart, Lung, and Blood Institute, National Institute of Health, Rockville, MD USA; 432 Public Health Division, An-Najah National University, Nablus, Palestine; 433 Institute of Molecular Biology and Biotechnology (IMBB), The University of Lahore, Lahore, Pakistan; 434 Research Centre for Health Sciences (RCHS), The University of Lahore, Lahore, Pakistan; 435 Department of Clinical Sciences, Al-Quds University, Ajman, United Arab Emirates; 436 Independent Consultant, Karachi, Pakistan; 437 Department of Pathology and Laboratory Medicine, Northwell Health, New York City, NY USA; 438 Amity Institute of Public Health, Amity University, Noida, India; 439 Department of Ophthalmology, Harvard Medical School, Boston, MA USA; 440 Ophthalmic Research Center (ORC), Shahid Beheshti University of Medical Sciences, Tehran, Iran; 441 Department of Veterinary Public Health and Preventive Medicine, Usmanu Danfodiyo University, Sokoto, Sokoto, Nigeria; 442 Center for Environmental and Respiratory Health Research, University of Oulu, Oulu, Finland; 443 National Institute of Environmental Health Sciences, National Health Research Institutes, Miaoli, Taiwan, ROC; 444 Department of Public Health Dentistry, Krishna Vishwa Vidyapeeth (Deemed to be University), Karad, India; 445 Department of Medical-Surgical Nursing, Mazandaran University of Medical Sciences, Sari, Iran; 446 Department of Nursing and Health Sciences, Flinders University, Adelaide, SA Australia; 447 Department of Pediatrics and Child Health Nursing, Dilla University, Dilla, Ethiopia; 448 Unit of Basic Medical Sciences, University of Khartoum, Khartoum, Sudan; 449 Department of Medical Microbiology and Infectious Diseases, Erasmus University, Rotterdam, The Netherlands; 450 Family, Health Promotion, and Life Course Department, Pan American Health Organization, Bogota, Colombia; 451 School of Medicine, University of Alabama at Birmingham, Birmingham, AL USA; 452 Department of Medicine Service, US Department of Veterans Affairs (VA), Birmingham, AL USA; 453 Department of Radiodiagnosis, All India Institute of Medical Sciences, Bathinda, India; 454 Department of Ophthalmology, Hywel Dda University Health Board, Llanelli, UK; 455 Department of Nursing, Dire Dawa University, Dire Dawa, Ethiopia; 456 Directive Board, Associação de Profissionais Licenciados de Optometria (Association of Licensed Optometry Professionals), Linda-a-Velha, Portugal; 457 Division of Community Medicine, International Medical University, Kuala Lumpur, Malaysia; 458 Federal Research Institute for Health Organization and Informatics of the Ministry of Health (FRIHOI), Moscow, Russia; 459 Soonchunhyang University, Vision Research Foundation, Cheonan-si, South Korea; 460 Nursing Professional Education Study Program, University Halu Oleo, Kendari, Indonesia; 461 Department of Medical Informatics, Mashhad University of Medical Sciences, Mashhad, Iran; 462 Clinical Research Development Unit, Mashhad University of Medical Sciences, Mashhad, Iran; 463 Department of Clinical Pharmacy, Mekelle University, Mekelle, Ethiopia; 464 Pediatric Intensive Care Unit, King Saud University, Riyadh, Saudi Arabia; 465 Outpatient Department, Wollega University, Bedele town, Ethiopia; 466 Department of Public Health, Wollega University, Nekemte, Ethiopia; 467 Faculty of Public Health, Universitas Sam Ratulangi, Manado, Indonesia; 468 Department of Public Health, Arba Minch College of Health Sciences, Arba Minch, Ethiopia; 469 Department of Medicine, University of Crete, Heraklion, Greece; 470 Department of Nursing, Aksum University, Aksum, Ethiopia; 471 Department of Physiology, East Carolina University, Greenville, NC USA; 472 Department of Epidemiology and Biostatistics, Haramaya University, Harar, Ethiopia; 473 Department of Pharmacology, All India Institute of Medical Sciences, Deoghar, India; 474 Department of Public Health, East Carolina University, Greenville, NC USA; 475 College of Public Health, Temple University, Philadelphia, PA USA; 476 Medical Genomics Research Department, King Abdullah International Medical Research Center, Riyadh, Saudi Arabia; 477 Division of Surgery and Interventional Science, University College London, London, UK; 478 Urmia University of Medical Sciences, Urmia, Iran; 479 Department of Cardiovascular Sciences, Katholieke Universiteit Leuven (University of Leuven), Leuven, Belgium; 480 Department of Public Health, Debre Markos University, Debre Markos, Ethiopia; 481 Department of Ophthalmology Research, Queen Mamohato Memorial Hospital, Maseru, Lesotho; 482 Ophthalmology Unit, Bahir Dar University, Bahirdar, Ethiopia; 483 School of Public Health, Zhejiang University, Zhejiang, China; 484 Department of Public Health Science, Fred Hutchinson Cancer Research Center, Seattle, WA USA; 485 China Center for Health Development Studies, Peking University, Beijing, China; 486 Center for the Study of Aging and Human Development, Duke University, Durham, NC USA; 487 The Russell H. Morgan Department of Radiology and Radiological Science, Johns Hopkins University, Baltimore, MD USA; 488 Department of Health Management, Süleyman Demirel Üniversitesi (Süleyman Demirel University), Isparta, Türkiye; 489 Department of Pharmacology, Bahir Dar University, Bahir Dar, Ethiopia; 490 Pharmacy Department, Alkan Health Science, Business and Technology College, Bahir Dar, Ethiopia; 491 Department of Pediatrics, Kyung Hee University, Seoul, South Korea; 492 Department of Neuropsychopharmacology, National Center of Neurology and Psychiatry, Kodaira, Japan; 493 Department of Public Health, Juntendo University, Tokyo, Japan; 494 Department of Epidemiology and Biostatistics, Wuhan University, Wuhan, China; 495 Department of Bioengineering and Therapeutic Sciences, University of California San Francisco, San Francisco, CA USA; 496 Addictology Department, Russian Medical Academy of Continuous Professional Education, Moscow, Russia; 497 College of Traditional Chinese Medicine, Hebei University, Baoding, China; 498 Department of Ophthalmology, Iran University of Medical Sciences, Tehran, Iran; 499 Department of Biochemistry and Pharmacogenomics, Medical University of Warsaw, Warsaw, Poland; 500 Department of Anatomy, Addis Ababa University, Addis Ababa, Ethiopia; 501 Department of Nursing, Yasuj University of Medical Sciences, Yasuj, Iran

**Keywords:** Epidemiology, Lens diseases

Correction to: *Eye* 10.1038/s41433-024-02961-1, published online 09 March 2024

The original online version of this article was revised. First of all, the author list has been corrected from “Konrad Pesudovs, Van Charles Lansingh, John H. Kempen, Ian Tapply, Arthur G. Fernandes, Maria V. Cicinelli, Alessandro Arrigo, Nicolas Leveziel, Paul Svitil Briant, Theo Vos, Serge Resnikoff, Hugh R. Taylor, Tabassom Sedighi, Seth Flaxman, Jaimie Steinmetz, Rupert R. A. Bourne, Vision Loss Expert Group of the Global Burden of Disease Study and the GBD 2019 Blindness and Vision Impairment Collaborators” to include only the following institutional authors “Vision Loss Expert Group of the Global Burden of Disease Study and the GBD 2019 Blindness and Vision Impairment Collaborators”. The list of the individual authors and affiliations now appear at the end of the original paper.

Furthermore, the following Article Note has been added: “These authors contributed equally: Jaimie Steinmetz and Rupert R. A. Bourne. These authors share the last authorship: Seth Flaxman and Jaimie Steinmetz.”

The title of Table 1 has been corrected from “Number of people (mean [95% UI]) with blindness (presenting visual acuity <3/60) or MSVI (presenting visual acuity <6/18, ≥3/60) due to Cataract, the age-standardized prevalence (%) in people of all ages and aged ≥50 years (mean [95% UI]), and the percentage of all blindness or MSVI attributed to Cataract (95% UI) in world regions in 2020” to “Number of people of all ages (mean [95% UI]) with blindness (presenting visual acuity <3/60) or MSVI (presenting visual acuity <6/18, ≥3/60) due to Cataract, the age-standardized prevalence (%) in people aged ≥50 years (mean [95% UI]), and the percentage of all blindness or MSVI attributed to Cataract in people aged ≥50 years (95% UI) in world regions in 2020”.

The following Data Availability statement was added: “Data sources for the Global Vision Database are listed at the following weblink http://www.anglia.ac.uk/verigbd. Fully disaggregated data is not available publicly due to data sharing agreements with some principal investigators yet requests for summary data can be made to the corresponding author.”

Furthermore, the information in the “Funding” section was incomplete. The following information was added: “This study was funded by Brien Holden Vision Institute, Fondation Thea, Fred Hollows Foundation, Bill & Melinda Gates Foundation, Lions Clubs International Foundation (LCIF), Sightsavers International, and University of Heidelberg.”

The “Competing interests” section has been corrected from “The authors declare no competing interests” to the following detailed information:

GBD 2019 Blindness and Vision Impairment Collaborators

Declarations

N S Bayileyegn reports participation on a Data Safety Monitoring Board or Advisory Board with Jimma University, and leadership or fiduciary roles in board, society, committee or advocacy groups, paid or unpaid with Jimma University as a discipline committee member; outside the submitted work. S Bhaskar reports grants or contracts from the Japan Society for the Promotion of Science (JSPS), JSPS International Fellowship, Japanese Ministry of Education, Culture, Sports, Science and Technology (MEXT), the Australian Academy of Science, Grant-in-Aid for Scientific Research (KAKENHI); leadership or fiduciary roles in board, society, committee or advocacy groups, paid or unpaid with Rotary District 9675 as the District Chair of Diversity, Equity, and Inclusion; the Global Health & Migration Hub Community and the Global Health Hub Germany (Berlin, Germany) as the Chair and Manager; PLOS One, BMC Neurology, Frontiers In Neurology, Frontiers in Stroke, Frontiers in Public Health and BMC Medical Research Methodology as an Editorial Board Member; and with the College of Reviewers, Canadian Institutes of Health Research (CIHR), and the Government of Canada as a Member; outside the submitted work. X Dai reports support for the present manuscript from the Institute for Health Metrics and Evaluation and the University of Washington. M Cenderadewi reports grants or contracts from James Cook University (International Research Training Program Scholarship for doctoral study), and support for attending meetings and travel from James Cook University; all outside the submitted work. M Foschi reports consulting fees from Roche; payment or honoraria for lectures, presentations, speakers bureaus, manuscript writing or educational events from Sanofi, Merck, and Novartis; support for attending meetings and travel from Novartis and Roche; leadership or fiduciary roles in board, society, committee or advocacy groups, paid or unpaid with MSBase as a scientific leadership board member, and Cochrane Review Group for Multiple Sclerosis and other rate diseases of the CNS as a member; all outside the submitted work. F Ghassemi reports support for the present manuscript from medical writing. B N G Goulart reports stock or stock options with Bristo Myers-Squibb and Pfizer; outside the submitted work. V B Gupta reports grants or contracts from the National Health and Medical Research Council (NHMRC); outside the submitted work. S Hallaj reports support for the present manuscript from the National Institute of Health, Bridge to AI common fund (grant number: OT2 OD032644). I M Ilic reports support for the present manuscript from the Ministry of Education, Science and Technological development, Republic of Serbia (project No 175042, 2011-2023). S Islam reports support for the present manuscript from the National Health and Medical Research Council (NHMRC) Investigator Grant and the Heart Foundation Vanguard Grant. J H Kempen reports support for the present manuscript from Sight for Souls and Mass Eye and Ear Global Surgery Program; and leadership or fiduciary roles in board, society, committee or advocacy groups, paid or unpaid with Sight for Souls as the President. K Krishan reports other non-financial support from the UGC Centre of Advanced Study, CAS II, awarded to the Department of Anthropology, Panjab University (Chandigarh, India); outside the submitted work. O P Kurmi reports grants or contracts from the British Council India paid to Coventry University; outside the submitted work. V C Lansingh reports consulting fees from HelpMeSee, and financial support for attending meetings and travel from HelpMeSee; outside the submitted work. J L Leasher leadership or fiduciary roles in board, society, committee or advocacy groups, unpaid with the National Eye Institute as a member and the National Eye Health Education Program as a planning committee member; outside the submitted work. M Lee reports support for the present manuscript from the Ministry of Education of the Republic of Korea, and the National Research Foundation of Korea (NRF-2021R1I1A4A01057428) and Bio-convergence Technology Education Program through the Korea Institute for Advancement Technology (KIAT) funded by the Ministry of Trade, Industry and Energy (No. P0017805). C McAlinden reports grants or contracts from the Welsh Government on the following study: Feasibility of an alternative pathway for hospital referrals from Diabetic Eye Screening Wales (DESW) for people suspected with sight-threatening diabetic eye disease (diabetic maculopathy). No funds will be received from the author’s institution or personally related to this study. Any work conducted as part of this study is as an unpaid collaborator; consulting fees from Acufocus (Irvine, California, USA), Atia Vision (Campbell, California, USA), Bausch and Lomb (Bridgewater, New Jersey, USA), BVI / PhysIOL (Liège, Belgium), Coopervision (Pleasanton, California, USA), Cutting Edge (Labége, France), Fudan University (Fudan, China), Hoya (Frankfurt, Germany), Knowledge Gate Group (Copenhagen, Denmark), Johnson & Johnson Surgical Vision (Santa Ana, California, USA), Keio University (Tokyo, Japan), Ludwig-Maximilians-University (München, Germany), Medevise Consulting SAS (Strasbourg, France), Novartis (Basel, Switzerland), Ophtec BV (Groningen, The Netherlands), Sun Yat-sen University (Guangzhou, China), SightGlass vision (Menlo Park, California, USA), Science in Vision (Bend, Oregan, USA), SpyGlass (Aliso Viejo, California, USA), Targomed GmbH (Bruchsal, Germany), University of São Paulo (São Paulo, Brazil), and Vold Vision (Arkansas, USA); payment or honoraria for lectures, presentations, speakers bureaus, manuscript writing or educational events from Scope (Crawley, UK), Bausch and Lomb (Bridgewater, New Jersey, USA), and Thea pharmaceuticals (Clemont-Ferrand, France); support for attending meetings and/or travel from Royal College of Ophthalmologists (London, UK), Scope (Crawley, UK), Portuguese Society of Ophthalmology (Portugal), British Society of Refractive surgery (BSRS), Thea pharmaceuticals (Clemont-Ferrand, France), Bausch and Lomb (Bridgewater, New Jersey, USA); leadership or fiduciary roles in board, society, committee or advocacy groups, paid or unpaid with the British Society of Refractive Surgery (BSRS) as an unpaid council member, and the Royal College of Ophthalmologists (London, UK) as an unpaid PROM advisor; other financial interests from the Quality of Vision (QoV) Questionnaire tool, the Orthokeratology and Contact Lens Quality of Life Questionnaire (OCL-QoL), and paid peer reviews for Research Square; outside of the submitted work. S Nargus reports receipt of equipment, materials, drugs, medical writing, gifts or other services from Medical writing services; outside the submitted work. Y L Samodra and J H V Ticoalu report other financial or non-financial interests as co-founders of Benang Merah Research Center; outside the submitted work. J A Singh reports consulting fees from AstraZeneca, Crealta/Horizon, Medisys, Fidia, PK Med, Two labs Inc., Adept Field Solutions, Clinical Care options, Clearview healthcare partners, Putnam associates, Focus forward, Navigant consulting, Spherix, MedIQ, Jupiter Life Science, UBM LLC, Trio Health, Medscape, WebMD, Practice Point communications, and the National Institutes of Health and the American College of Rheumatology; payment or honoraria for lectures, presentations, speakers bureaus, manuscript writing or educational events from the speaker’s bureau of Simply Speaking; support for attending meetings from OMERACT as a member of the steering committee; participation on an Advisory Committee with the FDA Arthritis Advisory Committee; leadership or fiduciary roles in board, society, committee or advocacy groups, paid or unpaid as a past steering committee member of the OMERACT, an international organization that develops measures for clinical trials and receives arms length funding from 12 pharmaceutical companies, Co-Chair of the Veterans Affairs Rheumatology Field Advisory Committee, and the editor and Director of the UAB Cochrane Musculoskeletal Group Satellite Center on Network Meta-analysis; stock or stock options in Atai Life Sciences, Kintara Therapeutics, Intelligent Biosolutions, Acumen Pharmaceutical, TPT Global Tech, Vaxart Pharmaceuticals, Atyu Biopharma, Adaptimmune Therapeutics, GeoVax Labs, Pieris Pharmaceuticals, Enzolytics Inc., Seres Therapeutics, Tonix Pharmaceuticals Holding Corp., and Charlotte’s Web Holdings, Inc, as well as previously owned stock options in Amarin, Viking and Moderna Pharmaceuticals; outside the submitted work. E Skiadaresi reports consulting fees from Bayer (Leverkusen, Germany), Novartis (Basel, Switzerland), Roche (Basel, Switzerland), Medevise Consulting SAS (Strasbourg, France); payment or honoraria for lectures, presentations, speakers bureaus, manuscript writing or educational events from Bayer (Leverkusen, Germany), Novartis (Basel, Switzerland), Roche (Basel, Switzerland); support for attending meetings and travel from Bayer (Leverkusen, Germany), Novartis (Basel, Switzerland), Roche (Basel, Switzerland); leadership or fiduciary roles in board, society, committee or advocacy groups, unpaid with the ATHENA Trial Steering Committee as the Chair; all outside the submitted work. J D Steinmetz reports support for the present manuscript from the Bill and Melinda Gates Foundation (IHME funding for GBD analyses). M Zielińska reports other financial interests as an AstraZeneca employee; outside the submitted work.

Vision Loss Expert Group of the Global Burden of Disease Study

Declarations

A Bron reports payment or honoraria for lectures, presentations, speakers bureaus, manuscript writing or educational events from Théa. M A Del Monte reports support for attending meetings and/or travel from the University of Michigan, and leadership or fiduciary roles in board, society, committee or advocacy groups, paid or unpaid as past president of Costenbader Society. D Friedman reports leadership or fiduciary role in other board, society, committee or advocacy group paid or unpaid with Orbis International as member of board of governors (no payment). J M Furtado reports consulting fees from Pan American Health Organization and from Lions Club International Foundation. M E Hartnett reports support for the present manuscript (e.g., funding, provision of study materials, medical writing, article processing charges, etc.) from Michael F. Marmor, M.D. Professor of Retinal Science and Disease as endowment to support salary, grants or contracts from any entity (from National Eye Institute R01 EY017011 and National Eye Institute R01 EY015130) as partial salary support, patents planned, issued or pending (WO2015123561A2 and WO2021062169A1), and leadership or fiduciary role in other board, society, committee or advocacy group, paid or unpaid with Jack McGovern Coats’ Disease Foundation and as director of Women’s Eye Health and Macular Society Grant Review Chair. J H Kempen reports support for the present manuscript (e.g., funding, provision of study materials, medical writing, article processing charges, etc.) from Sight for Souls and from Mass Eye and Ear Global Surgery Program (both as general support of salary), and leadership or fiduciary role in other board, society, committee or advocacy group, paid or unpaid with Sight for Souls (as president). J E Kim reports consulting fees from Genentech/Roche, DORC, Notal Vision and Outlook Therapeutics (all as payment to J E Kim); participation on a Data Safety Monitoring Board or Advisory Board with Allergan, Amgen, Apellis, Bausch&Lomb, Clearside, Coherus, Novartis and Regeneron (all as participation on advisory board); leadership or fiduciary role in other borad, society, committee or advocacy group, paid or unpaid, with AAO, APRIS, ASRS, Macular Society and NAEVR/AEVR (all unpaid); and receipt of equipment, materials, drugs, medical writing, gifts or other services from Clearside and Genentech/Roche (both for medical writing). V C Lansingh reports consulting fees from HelpMeSee (as an employee), and support for attending meetings and/or travel from HelpMeSee (pay airfare and hotel). J Leasher reports leadership or fiduciary role in other board, society, committee or advocacy group, paid or unpaid with National Eye Institute (as a member) and National Eye Health Education Program planning committee (unpaid). M Nowak reports participation on a Data Safety Monitoring Board or Advisory Board with Vision Express Co. Poland as the chairman of medical advisory board of Vision Express Co. Poland. P Ramulu reports grants or contracts from National Institute of Health and Perfuse Therapeutics, and consulting fees from Alcon and W. L. Gore. F Topouzis reports grants or contracts from Théa, Omikron, Pfizer, Alcon, Abbvie and Bayer (all paid to Institution), consulting fees from Omikron, Théa and Bausch & Lomb (all paid to Topouzis), payment or honoraria for lectures, presentations, speakers bureaus, manuscript writing or educational events from Omikron (paid to Topouzis), Abbvie and Roche (both paid to Institute), and leadership or fiduciary role in other board, society, committee or advocacy group, paid or unpaid with European Glaucoma Society (as president), Greek Glaucoma Society (as president) and Board of Governors, World Glaucoma Association (all unpaid).

Finally, the section “Author Contributions” has been added and an Appendix with more detailed information for individual author contributions has been included in the form of electronic supplementary material.

The original article has been corrected.

### Supplementary information

Appendix

