# Supplementary material for: Global estimates on the number of people blind or visually impaired by cataract: a meta-analysis from 2000 to 2020
Source: Eye (Lond). 2024 Mar 9;38(11):2156–72. doi: 10.1038/s41433-024-02961-1 (PMC11269584; doi:10.1038/s41433-024-02961-1)
Supplement: Supplementary file 1 — Supplementary File [file 41433_2024_2961_MOESM1_ESM.docx]

**Appendix: Contributions by Authors**

# GBD 2019 Blindness and Vision Impairment Collaborators

## Providing data or critical feedback on data sources

Yohannes Habtegiorgis Abate, Mozhan Abdollahi, Ayele Mamo Abebe, Richard Gyan Aboagye, Hiwa Abubaker Ali, Tadele Girum Girum Adal, Mesafint Molla Adane, Qorinah Estiningtyas Sakilah Adnani, Bright Opoku Ahinkorah, Sajjad Ahmad, Ali Ahmadi, Ayman Ahmed, Haroon Ahmed, Abid Ali, Syed Shujait Shujait Ali, Awais Altaf, Hubert Amu, Sofia Androudi, Rodrigo Anguita, Saeid Anvari, Francis Appiah, Jalal Arabloo, Reza Arefnezhad, Tahira Ashraf, Seyyed Shamsadin Athari, Bantalem Tilaye Tilaye Atinafu, Maha Moh'd Wahbi Atout, Alok Atreya, Ahmed Y Azzam, Sara Bagherieh, Atif Amin Baig, Biswajit Banik, Mainak Bardhan, Nebiyou Simegnew Bayileyegn, Akshaya Srikanth Bhagavathula, Sonu Bhaskar, Jasvinder Singh Bhatti, Mukharram Bikbov, Niloufar Bineshfar, Rupert Bourne, Tasanee Braithwaite, Paul Svitil Briant, Florentino Luciano Caetano dos Santos, Muthia Cenderadewi, Vijay Kumar Chattu, Hitesh Chopra, Dinh-Toi Chu, Maria Vittoria Cicinelli, João M Coelho, Natália Cruz-Martins, Xiaochen Dai, Ana Maria Dascalu, Maedeh Dastmardi, Awoke Masrie Asrat Derese, Nikolaos Dervenis, Thanh Chi Do, Thao Huynh Phuong Do, Francisco Winter dos Santos Figueiredo, Hisham Atan Edinur, Joshua R Ehrlich, Michael Ekholuenetale, Temitope Cyrus Ekundayo, Iman El Sayed, Mohammad Hassan Emamian, Adeniyi Francis Fagbamigbe, Ayesha Fahim, Hossein Farrokhpour, Ali Fatehizadeh, Alireza Feizkhah, Lorenzo Ferro Desideri, Brhane Gebremariam, Sherief Ghozy, Mahaveer Golechha, Pouya Goleij, Bárbara Niegia Garcia Goulart, Zewdie Gudisa, Sapna Gupta, Veer Bala Gupta, Vivek Kumar Gupta, Arvin Haj-Mirzaian, Aram Halimi, Demisu Zenbaba Heyi, Sung Hwi Hong, Mehdi Hosseinzadeh, John J Huang, Hong-Han Huynh, Segun Emmanuel Ibitoye, Sheikh Mohammed Shariful Islam, Ammar Abdulrahman Jairoun, Shubha Jayaram, Charity Ehimwenma Joshua, Vidya Kadashetti, Sagarika Kamath, Himal Kandel, Rami S Kantar, Soujanya Kaup, Gbenga A Kayode, Yousef Saleh Khader, Himanshu Khajuria, Rovshan Khalilov, Mahalaqua Nazli Khatib, Adnan Kisa, Soewarta Kosen, Kewal Krishan, Mukhtar Kulimbet, Om P Kurmi, Chandrakant Lahariya, Van Charles Lansingh, Janet L Leasher, Munjae Lee, Seung Won Lee, Stephen S Lim, Preetam Bhalchandra Mahajan, Sandeep B Maharaj, Razzagh Mahmoudi, Kashish Malhotra, Tauqeer Hussain Mallhi, Roy Rillera Marzo, Andrea Maugeri, Colm McAlinden, Tesfahun Mekene Meto, Le Huu Nhat Minh, Awoke Misganaw, Manish Mishra, Soheil Mohammadi, Mustapha Mohammed, Ali H Mokdad, Mohammad Ali Moni, Maryam Moradi, Rohith Motappa, Admir Mulita, Christopher J L Murray, Ganesh R Naik, Shumaila Nargus, Zuhair S Natto, Mohammad Negaresh, Dang H Nguyen, Phat Tuan Nguyen, Van Thanh Nguyen, Robina Khan Niazi, Osaretin Christabel Okonji, Andrew T Olagunju, Matthew Idowu Olatubi, Uchechukwu Levi Osuagwu, Mayowa O Owolabi, Jagadish Rao Padubidri, Ashok Pandey, Jay Patel, Shrikant Pawar, Konrad Pesudovs, Hoang Tran Pham, Nguyen Khoi Quan, Fakher Rahim, Vafa Rahimi-Movaghar, Sathish Rajaa, Shakthi Kumaran Ramasamy, Annisa Utami Rauf, Salman Rawaf, Serge Resnikoff, Koushik Roy Pramanik, Siamak Sabour, Basema Saddik, Umar Saeed, Fatemeh Saheb Sharif-Askari, Mohammad Ali Sahraian, Sara Samadzadeh, Vijaya Paul Samuel, Abdallah M Samy, Tabassom Sedighi, Yashendra Sethi, Allen Seylani, Amira A Shaheen, Masood Ali Shaikh, Sunder Sham, Mohammed Shannawaz, Maryam Shayan, Aminu Shittu, K M Shivakumar, Juan Carlos Silva, Jasvinder A Singh, Paramdeep Singh, Eirini Skiadaresi, Yonatan Solomon, Chandrashekhar T Sreeramareddy, Jaimie D Steinmetz, Mohana Devi Subramaniam, Ian Tapply, Hugh R Taylor, Temesgen Mohammed Toma, Guesh Mebrahtom Tsegay, Sree Sudha Ty, Muhammad Umair, Tungki Pratama Umar, Jef Van den Eynde, Theo Vos, Stephanie Louise Watson Watson, Tewodros Eshete Wonde, Guadie Sharew Wondimagegn, Hong Xiao, Dong Keon Yon, Naohiro Yonemoto, Chuanhua Yu, Mikhail Sergeevich Zastrozhin, Magdalena Zielińska, Yossef Teshome Zikarg, and Mohammad Zoladl.

## Developing methods or computational machinery

Mozhan Abdollahi, Ayele Mamo Abebe, Hiwa Abubaker Ali, Qorinah Estiningtyas Sakilah Adnani, Ali Ahmadi, Hubert Amu, Ahmed Y Azzam, Akshaya Srikanth Bhagavathula, Rupert Bourne, Paul Svitil Briant, Kaleb Coberly, Xiaochen Dai, Maedeh Dastmardi, Awoke Masrie Asrat Derese, Thanh Chi Do, Ayesha Fahim, Ali Fatehizadeh, Alireza Feizkhah, Lorenzo Ferro Desideri, Sherief Ghozy, Aram Halimi, Mehdi Hosseinzadeh, Hong-Han Huynh, Rohollah Kalhor, Rovshan Khalilov, Mahalaqua Nazli Khatib, Adnan Kisa, Chandrakant Lahariya, Van Charles Lansingh, Razzagh Mahmoudi, Le Huu Nhat Minh, Hoda Mojiri-forushani, Ali H Mokdad, Mohammad Ali Moni, Admir Mulita, Christopher J L Murray, Dang H Nguyen, Phat Tuan Nguyen, Van Thanh Nguyen, Michal Ordak, Hoang Tran Pham, Umar Saeed, Abdallah M Samy, Jaimie D Steinmetz, Ian Tapply, Muhammad Umair, Theo Vos, Tewodros Eshete Wonde, Hanqing Zhao, and Yossef Teshome Zikarg.

## Providing critical feedback on methods or results

Yohannes Habtegiorgis Abate, Mohammad Abdollahi, Mozhan Abdollahi, Ayele Mamo Abebe, Olumide Abiodun, Richard Gyan Aboagye, Woldu Aberhe Abrha, Hiwa Abubaker Ali, Eman Abu-Gharbieh, Salahdein Aburuz, Tadele Girum Girum Adal, Mesafint Molla Adane, Isaac Yeboah Addo, Qorinah Estiningtyas Sakilah Adnani, Muhammad Sohail Afzal, Shahin Aghamiri, Bright Opoku Ahinkorah, Aqeel Ahmad, Sajjad Ahmad, Ali Ahmadi, Ayman Ahmed, Haroon Ahmed, Ahmad Samir Alfaar, Abid Ali, Syed Shujait Shujait Ali, Awais Altaf, Hubert Amu, Sofia Androudi, Rodrigo Anguita, Saeid Anvari, Anayochukwu Edward Anyasodor, Francis Appiah, Jalal Arabloo, Mosab Arafat, Akeza Awealom Asgedom, Tahira Ashraf, Seyyed Shamsadin Athari, Bantalem Tilaye Tilaye Atinafu, Maha Moh'd Wahbi Atout, Alok Atreya, Haleh Ayatollahi, Ahmed Y Azzam, Hassan Babamohamadi, Sara Bagherieh, Yogesh Bahurupi, Atif Amin Baig, Biswajit Banik, Mainak Bardhan, Saurav Basu, Kavita Batra, Nebiyou Simegnew Bayileyegn, Fatemeh Bazvand, Addisu Shunu Beyene, Devidas S Bhagat, Akshaya Srikanth Bhagavathula, Pankaj Bhardwaj, Sonu Bhaskar, Jasvinder Singh Bhatti, Mukharram Bikbov, Marina G Birck, Rupert Bourne, Tasanee Braithwaite, Paul Svitil Briant, Katrin Burkart, Yasser Bustanji, Zahid A Butt, Florentino Luciano Caetano dos Santos, Luis Alberto Cámera, Muthia Cenderadewi, Eeshwar K Chandrasekar, Vijay Kumar Chattu, Nitin Chitranshi, Hitesh Chopra, Dinh-Toi Chu, João M Coelho, Natália Cruz-Martins, Omid Dadras, Xiaochen Dai, Subasish Das, Ana Maria Dascalu, Mohsen Dashti, Maedeh Dastmardi, Berecha Hundessa Demessa, Biniyam Demisse, Awoke Masrie Asrat Derese, Nikolaos Dervenis, Vinoth Gnana Chellaiyan Devanbu, Thanh Chi Do, Thao Huynh Phuong Do, Francisco Winter dos Santos Figueiredo, Arkadiusz Marian Dziedzic, Hisham Atan Edinur, Ferry Efendi, Joshua R Ehrlich, Michael Ekholuenetale, Temitope Cyrus Ekundayo, Iman El Sayed, Muhammed Elhadi, Mohammad Hassan Emamian, Mehdi Emamverdi, Adeniyi Francis Fagbamigbe, Ayesha Fahim, Hossein Farrokhpour, Ali Fatehizadeh, Lorenzo Ferro Desideri, Getahun Fetensa, Florian Fischer, Matteo Foschi, Kayode Raphael Fowobaje, Abhay Motiramji Gaidhane, Aravind P Gandhi, Miglas W W Gebregergis, Mesfin Gebrehiwot, Brhane Gebremariam, Urge Gerema, Fariba Ghassemi, Sherief Ghozy, Mahaveer Golechha, Bárbara Niegia Garcia Goulart, Shi-Yang Guan, Sapna Gupta, Veer Bala Gupta, Vivek Kumar Gupta, Aram Halimi, Shahin Hallaj, Samer Hamidi, Mehdi Harorani, Hamidreza Hasani, Demisu Zenbaba Heyi, Nguyen Quoc Hoan, Ramesh Holla, Sung Hwi Hong, Mehdi Hosseinzadeh, Chengxi Hu, Hong-Han Huynh, Segun Emmanuel Ibitoye, Irena M Ilic, Mustapha Immurana, Md. Rabiul Islam, Sheikh Mohammed Shariful Islam, Chidozie C D Iwu, Louis Jacob, Ammar Abdulrahman Jairoun, Shubha Jayaram, Har Ashish Jindal, Mohammad Jokar, Nitin Joseph, Charity Ehimwenma Joshua, Vidya Kadashetti, Rohollah Kalhor, Sagarika Kamath, Himal Kandel, Rami S Kantar, Ibraheem M Karaye, Soujanya Kaup, Navjot Kaur, Rimple Jeet Kaur, Gbenga A Kayode, John H Kempen, Himanshu Khajuria, Rovshan Khalilov, Mahalaqua Nazli Khatib, Adnan Kisa, Ai Koyanagi, Kewal Krishan, Nithin Kumar, Om P Kurmi, Chandrakant Lahariya, Tuo Lan, Iván Landires, Van Charles Lansingh, Janet L Leasher, Seung Won Lee, Wei-Chen Lee, Stephen S Lim, Preetam Bhalchandra Mahajan, Alireza Mahmoudi, Razzagh Mahmoudi, Kashish Malhotra, Tauqeer Hussain Mallhi, Vahid Mansouri, Emmanuel Manu, Roy Rillera Marzo, Andrea Maugeri, Colm McAlinden, Wondwosen Mebratu, Tesfahun Mekene Meto, Yang Meng, Abera M Mersha, Tomislav Mestrovic, Le Huu Nhat Minh, Awoke Misganaw, Manish Mishra, Sanjeev Misra, Nouh Saad Mohamed, Soheil Mohammadi, Mustapha Mohammed, Ali H Mokdad, Hossein Molavi Vardanjani, Mohammad Ali Moni, Fateme Montazeri, Maryam Moradi, Rohith Motappa, Admir Mulita, Christopher J L Murray, Ganesh R Naik, Gurudatta Naik, Shumaila Nargus, Zuhair S Natto, Hadush Negash, Dang H Nguyen, Phat Tuan Nguyen, Van Thanh Nguyen, Robina Khan Niazi, Osaretin Christabel Okonji, Andrew T Olagunju, Matthew Idowu Olatubi, Michal Ordak, Uchechukwu Levi Osuagwu, Nikita Otstavnov, Mayowa O Owolabi, Jagadish Rao Padubidri, Ashok Pandey, Georgios D Panos, Shahina Pardhan, Seoyeon Park, Jay Patel, Shrikant Pawar, Prince Peprah, Konrad Pesudovs, Ionela-Roxana Petcu, Hoang Tran Pham, Mohsen Pourazizi, Nguyen Khoi Quan, Fakher Rahim, Vafa Rahimi-Movaghar, Sathish Rajaa, Shakthi Kumaran Ramasamy, Premkumar Ramasubramani, Shubham Ranjan, Mohammad-Mahdi Rashidi, Salman Rawaf, Elrashdy Moustafa Mohamed Redwan, Serge Resnikoff, Priyanka Roy, Koushik Roy Pramanik, Zahra Saadatian, Siamak Sabour, Basema Saddik, Umar Saeed, Sare Safi, Sher Zaman Safi, Fatemeh Saheb Sharif-Askari, Mohammad Ali Sahraian, Joseph W Sakshaug, Mohamed A Saleh, Sara Samadzadeh, Yoseph Leonardo Samodra, Vijaya Paul Samuel, Abdallah M Samy, Sabyasachi Senapati, Yashendra Sethi, Seyed Arsalan Seyedi, Amira A Shaheen, Samiah Shahid, Moyad Jamal Shahwan, Masood Ali Shaikh, Muhammad Aaqib Shamim, Mohammed Shannawaz, Bereket Beyene Shashamo, Maryam Shayan, Aminu Shittu, Ivy Shiue, K M Shivakumar, Seyed Afshin Shorofi, Migbar Mekonnen Sibhat, Emmanuel Edwar Siddig, Jasvinder A Singh, Paramdeep Singh, Eirini Skiadaresi, Yonatan Solomon, Chandrashekhar T Sreeramareddy, Jaimie D Steinmetz, Seyyed Mohammad Tabatabaei, Ian Tapply, Birhan Tsegaw Taye, Hugh R Taylor, Gebrehiwot Teklay, Mohamad-Hani Temsah, Jansje Henny Vera Ticoalu, Temesgen Mohammed Toma, Aristidis Tsatsakis, Guesh Mebrahtom Tsegay, Munkhtuya Tumurkhuu, Biruk Shalmeno Tusa, Chukwudi S Ubah, Muhammad Umair, Tungki Pratama Umar, Rohollah Valizadeh, Jef Van den Eynde, Theo Vos, Stephanie Louise Watson Watson, Tewodros Eshete Wonde, Guadie Sharew Wondimagegn, Hong Xiao, Yao Yao, Iman Yazdani Nia, Arzu Yiğit, Yazachew Yismaw, Dong Keon Yon, Naohiro Yonemoto, Yuyi You, Chuanhua Yu, Mikhail Sergeevich Zastrozhin, Hanqing Zhao, Magdalena Zielińska, Yossef Teshome Zikarg, and Mohammad Zoladl.

## Drafting the work or revising it critically for important intellectual content

Yohannes Habtegiorgis Abate, Mozhan Abdollahi, Ayele Mamo Abebe, Olumide Abiodun, Hasan Abualruz, Eman Abu-Gharbieh, Salahdein Aburuz, Mesafint Molla Adane, Isaac Yeboah Addo, Qorinah Estiningtyas Sakilah Adnani, Muhammad Sohail Afzal, Bright Opoku Ahinkorah, Ali Ahmadi, Ayman Ahmed, Haroon Ahmed, Ahmad Samir Alfaar, Abid Ali, Awais Altaf, Hubert Amu, Rodrigo Anguita, Abhishek Anil, Anayochukwu Edward Anyasodor, Jalal Arabloo, Brhane Berhe Aregawi, Alessandro Arrigo, Akeza Awealom Asgedom, Seyyed Shamsadin Athari, Bantalem Tilaye Tilaye Atinafu, Maha Moh'd Wahbi Atout, Alok Atreya, Ahmed Y Azzam, Hassan Babamohamadi, Sara Bagherieh, Mainak Bardhan, Saurav Basu, Addisu Shunu Beyene, Akshaya Srikanth Bhagavathula, Sonu Bhaskar, Jasvinder Singh Bhatti, Mukharram Bikbov, Marina G Birck, Veera R Bitra, Rupert Bourne, Tasanee Braithwaite, Yasser Bustanji, Florentino Luciano Caetano dos Santos, Vera L A Carneiro, Muthia Cenderadewi, Eeshwar K Chandrasekar, Vijay Kumar Chattu, Hitesh Chopra, Dinh-Toi Chu, João M Coelho, Natália Cruz-Martins, Ana Maria Dascalu, Mohsen Dashti, Maedeh Dastmardi, Berecha Hundessa Demessa, Biniyam Demisse, Diriba Dereje, Awoke Masrie Asrat Derese, Nikolaos Dervenis, Vinoth Gnana Chellaiyan Devanbu, Thanh Chi Do, Francisco Winter dos Santos Figueiredo, Iman El Sayed, Muhammed Elhadi, Mohammad Hassan Emamian, Mehdi Emamverdi, Azin Etemadimanesh, Adeniyi Francis Fagbamigbe, Ayesha Fahim, Ali Fatehizadeh, Lorenzo Ferro Desideri, Florian Fischer, Ali Forouhari, Matteo Foschi, Aravind P Gandhi, Miglas W W Gebregergis, Mesfin Gebrehiwot, Brhane Gebremariam, Fariba Ghassemi, Sherief Ghozy, Bárbara Niegia Garcia Goulart, Shi-Yang Guan, Sapna Gupta, Veer Bala Gupta, Vivek Kumar Gupta, Arvin Haj-Mirzaian, Aram Halimi, Shahin Hallaj, Hamidreza Hasani, Demisu Zenbaba Heyi, Nguyen Quoc Hoan, Ramesh Holla, Sung Hwi Hong, Hong-Han Huynh, Segun Emmanuel Ibitoye, Irena M Ilic, Mustapha Immurana, Md. Rabiul Islam, Sheikh Mohammed Shariful Islam, Chidozie C D Iwu, Louis Jacob, Ammar Abdulrahman Jairoun, Manthan Dilipkumar Janodia, Shubha Jayaram, Har Ashish Jindal, Nitin Joseph, Vidya Kadashetti, Laleh R Kalankesh, Sagarika Kamath, Himal Kandel, Rami S Kantar, Hengameh Kasraei, Soujanya Kaup, Navjot Kaur, Rimple Jeet Kaur, Gbenga A Kayode, John H Kempen, Yousef Saleh Khader, Himanshu Khajuria, Rovshan Khalilov, Mahalaqua Nazli Khatib, Adnan Kisa, Ai Koyanagi, Kewal Krishan, Mukhtar Kulimbet, Chandrakant Lahariya, Iván Landires, Van Charles Lansingh, Janet L Leasher, Seung Won Lee, Julie-Anne Little, Preetam Bhalchandra Mahajan, Razzagh Mahmoudi, Tauqeer Hussain Mallhi, Vahid Mansouri, Emmanuel Manu, Roy Rillera Marzo, Andrea Maugeri, Colm McAlinden, Wondwosen Mebratu, Tomislav Mestrovic, Le Huu Nhat Minh, Awoke Misganaw, Soheil Mohammadi, Mustapha Mohammed, Hoda Mojiri-forushani, Ali H Mokdad, Mohammad Ali Moni, Fateme Montazeri, Maryam Moradi, Rohith Motappa, Parsa Mousavi, Christopher J L Murray, Shumaila Nargus, Zuhair S Natto, Biswa Prakash Nayak, Mohammad Negaresh, Hadush Negash, Dang H Nguyen, Phat Tuan Nguyen, Van Thanh Nguyen, Robina Khan Niazi, Osaretin Christabel Okonji, Andrew T Olagunju, Matthew Idowu Olatubi, Michal Ordak, Uchechukwu Levi Osuagwu, Nikita Otstavnov, Mayowa O Owolabi, Jagadish Rao Padubidri, Shahina Pardhan, Seoyeon Park, Jay Patel, Shrikant Pawar, Konrad Pesudovs, Ionela-Roxana Petcu, Alireza Peyman, Hoang Tran Pham, Mohsen Pourazizi, Nguyen Khoi Quan, Fakher Rahim, Vafa Rahimi-Movaghar, Mohammad Hifz Ur Rahman, Sathish Rajaa, Shubham Ranjan, Rama Shankar Rath, Annisa Utami Rauf, Salman Rawaf, Amirmasoud Rayati Damavandi, Elrashdy Moustafa Mohamed Redwan, Priyanka Roy, Koushik Roy Pramanik, Zahra Saadatian, Siamak Sabour, Basema Saddik, Umar Saeed, Sare Safi, Amene Saghazadeh, Fatemeh Saheb Sharif-Askari, Amirhossein Sahebkar, Mohammad Ali Sahraian, Sara Samadzadeh, Yoseph Leonardo Samodra, Vijaya Paul Samuel, Abdallah M Samy, Aswini Saravanan, Siddharthan Selvaraj, Farbod Semnani, Sabyasachi Senapati, Yashendra Sethi, Allen Seylani, Samiah Shahid, Moyad Jamal Shahwan, Muhammad Aaqib Shamim, Mohammed Shannawaz, Bereket Beyene Shashamo, K M Shivakumar, Seyed Afshin Shorofi, Migbar Mekonnen Sibhat, Emmanuel Edwar Siddig, Juan Carlos Silva, Jasvinder A Singh, Paramdeep Singh, Eirini Skiadaresi, Yonatan Solomon, Raúl A R C Sousa, Chandrashekhar T Sreeramareddy, Vladimir I Starodubov, Sri Susanty, Gebrehiwot Teklay, Mohamad-Hani Temsah, Dufera Rikitu Terefa, Jansje Henny Vera Ticoalu, Temesgen Mohammed Toma, Munkhtuya Tumurkhuu, Sree Sudha Ty, Chukwudi S Ubah, Muhammad Umair, Tungki Pratama Umar, Jef Van den Eynde, Theo Vos, Stephanie Louise Watson Watson, Tewodros Eshete Wonde, Guadie Sharew Wondimagegn, Yao Yao, Iman Yazdani Nia, Arzu Yiğit, Dong Keon Yon, Naohiro Yonemoto, Mikhail Sergeevich Zastrozhin, Hanqing Zhao, Makan Ziafati, Magdalena Zielińska, Yossef Teshome Zikarg, and Mohammad Zoladl.

## Managing the estimation or publications process

Ali Ahmadi, Awais Altaf, Ahmed Y Azzam, Maedeh Dastmardi, Thanh Chi Do, Ali Fatehizadeh, Lorenzo Ferro Desideri, Hong-Han Huynh, Soujanya Kaup, Mahalaqua Nazli Khatib, Chandrakant Lahariya, Van Charles Lansingh, Razzagh Mahmoudi, Le Huu Nhat Minh, Mustapha Mohammed, Ali H Mokdad, Maryam Moradi, Phat Tuan Nguyen, Van Thanh Nguyen, Konrad Pesudovs, Hoang Tran Pham, Nguyen Khoi Quan, Abdallah M Samy, Muhammad Umair, Jef Van den Eynde, Theo Vos, Tewodros Eshete Wonde, and Mikhail Sergeevich Zastrozhin.

Vision Loss Expert Group of the Global Burden of Disease Study

### Providing data or critical feedback on data sources

Alessandro Arrigo, Mukharram M Bikbov, Rupert R A Bourne, Tasanee Braithwaite, Alain Bron, Ching-Yu Cheng, Maria Vittoria Cicinelli, Monte A Del Monte, Joshua R Ehrlich, Leon B Ellwein, Arthur Fernandes, Seth Flaxman, David Friedman, João M Furtado, Gus Gazzard, Ronnie George, M Elizabeth Hartnett, Jost B Jonas, Rim Kahloun, John H Kempen, Moncef Khairallah, Rohit C Khanna, Judy E Kim, Van Charles Lansingh, Janet Leasher, Nicolas Leveziel, Julie-Anne Little, Kovin S Naidoo, Vinay Nangia, Michal Nowak, Konrad Pesudovs, Tunde Peto, Pradeep Ramulu, Serge Resnikoff, Tabassom Sedighi, Ian Tapply, Hugh Taylor, Fotis Topouzis, Miltiadis Tsilimbaris, Ya Xing Wang, Ningli Wang

### Developing methods or computational machinery

Rupert R A Bourne, Jost B Jonas, Van Charles Lansingh, Ian Tapply

### Providing critical feedback on methods or results

Alessandro Arrigo, Mukharram M Bikbov, Rupert R A Bourne, Tasanee Braithwaite, Monte A Del Monte, David Friedman, João M Furtado, M Elizabeth Hartnett, Jost B Jonas, Rim Kahloun, John H Kempen, Van Charles Lansingh, Julie-Anne Little, Konrad Pesudovs, Serge Resnikoff, Ian Tapply, Ningli Wang

### Drafting the work or revising it critically for important intellectual content

Alessandro Arrigo, Mukharram M Bikbov, Rupert R A Bourne, Tasanee Braithwaite, Monte A Del Monte, Ronnie George, M Elizabeth Hartnett, Jost B Jonas, John H Kempen, Van Charles Lansingh, Janet Leasher, Julie-Anne Little, Konrad Pesudovs

### Managing the estimation or publications process

Rupert R A Bourne, Jost B Jonas, Van Charles Lansingh, Konrad Pesudovs
